# NAD^+^ metabolism influences TGF-β–associated transcriptional programs and the gut microbiota in murine models of colitis

**DOI:** 10.1016/j.bbrep.2026.102752

**Published:** 2026-08-22

**Authors:** Hisayuki Amano, Akiyoshi Komuro, Kazushige Ota, Masahiko Honda, Kazuko Sakai, Kazuto Nishio, Takeshi Ueda, Hitoshi Okada

**Affiliations:** a Department of Biochemistry, Faculty of Medicine, Kindai University, Osaka, Japan; b Department of Genome Biology, Faculty of Medicine, Kindai University, Osaka, Japan

**Keywords:** NAD^+^ metabolism, TGF-β, Fibrosis, Inflammation, Microbiome

## Abstract

NAD^+^ metabolism plays an important role in maintaining cellular homeostasis and has been implicated in fibrosis-related diseases, including inflammatory bowel disease (IBD). However, the molecular and system-level mechanisms linking NAD^+^ metabolism to fibrosis remain incompletely understood.

Here, we investigated the effects of nicotinamide mononucleotide (NMN), a key NAD^+^ precursor, on transforming growth factor β (TGF-β)-associated transcriptional programs. In mouse embryonic fibroblasts, NMN partially restored a subset of TGF-β-associated transcriptional changes, including alterations in *Lgr5* and *Naprt* expression, and affected the expression of fibrosis-associated genes such as *Nt5e* and *Enpp1*, suggesting an association between NMN metabolism and TGF-β-associated transcriptional programs.

In a prophylactic DSS-induced mouse model of colitis, NMN supplementation was associated with similar transcriptional changes *in vivo*, including increased expression of regeneration-associated genes and reduced fibrosis- and immune-related pathways. Microbiome analysis revealed NMN-associated shifts in bacterial communities.

Long-term NMN administration was associated with altered colonic immune transcriptional signatures, including alterations in TGF-β-associated and IgA-related pathways, without detectable changes in B-cell or plasma-cell abundance. In a colitis-associated cancer model, NMN administration did not significantly affect tumor number but was associated with changes in TGF-β-related immune pathways within tumors.

These findings indicate that NMN supplementation is associated with changes in TGF-β-associated transcriptional and immunological networks across cellular, microbial, and tissue contexts, providing insights into the potential contribution of NAD^+^ metabolism in preventive approaches for fibrosis-related inflammatory conditions.

## Highlights

• NMN is associated with altered TGF-β-related transcriptional programs.
• NMN modifies colonic gene expression in experimental colitis.
• NMN is associated with changes in gut microbiota composition.
• Long-term NMN administration alters IgA-related immune pathways.
• NMN exhibits preventive effects in experimental colitis models.

## 1 Introduction

Inflammatory bowel diseases (IBD), including ulcerative colitis and Crohn's disease, are characterized by chronic inflammation and immune dysregulation [[1], [2], [3]]. Despite global improvements in hygiene and sanitation, the prevalence of IBD has increased with economic development, and these diseases are increasingly affecting elderly populations [[4], [5], [6], [7]]. This epidemiological shift underscores the multifactorial nature of IBD, involving not only genetic predisposition but also environmental factors such as aging and lifestyle. With the aging of the global population, the incidence of IBD is expected to increase, potentially leading to more long-term complications and associated malignancies. Notably, aging-associated alterations in cellular metabolism and immune regulation are increasingly recognized as critical modifiers of disease susceptibility and progression.

IBD pathology involves persistent immune cell infiltration and fibrosis in the intestinal mucosa [8]. Fibrosis, defined as the pathological accumulation of extracellular matrix proteins, arises from chronic inflammation and is influenced by genetic factors, aging, and lifestyle [9,10]. Patients with IBD are at increased risk of colitis-associated cancer (CAC), as sustained inflammation promotes neoplastic progression [11,12]. Although CAC accounts for a small proportion of colorectal cancer cases, its clinical outcomes are generally worse than those of sporadic colorectal cancer [11,13]. Additionally, IBD is strongly associated with dysbiosis, a disruption of normal gut microbiome homeostasis that impairs colonic barrier function and immune regulation, thereby contributing to fibrosis, inflammation, and tumorigenesis [[14], [15], [16]].

Transforming growth factor β (TGF-β) is a pleiotropic cytokine that exerts both anti-inflammatory and profibrotic effects [[17], [18], [19], [20]]. Under physiological conditions, this signaling is essential for tissue repair and regeneration. Aberrant TGF-β activation, however, induces excessive expression of profibrotic genes in fibroblasts, driving fibrosis [21]. Its signaling involves rapid nuclear translocation of the SMAD2/3/4 complex, leading to the transcription of primary response genes within hours, while secondary response genes are regulated indirectly or through epigenetic memory [22,23]. Both murine models and human studies have shown that TGF-β deficiency or loss-of-function leads to severe colitis, whereas appropriate modulation of this signaling can ameliorate fibrosis, highlighting its critical role in immune regulation and mucosal integrity [[24], [25], [26]]. Recent studies suggest that metabolic state, including NAD^+^ availability, can influence TGF-β signaling and its downstream transcriptional programs.

The gut microbiota interacts closely with TGF-β signaling [27]. Certain commensal bacteria, including *Clostridium clusters IV* and *XIVa* and *Bacteroides fragilis*, enhance TGF-β production by epithelial and dendritic cells. TGF-β promotes class-switch recombination in B cells to generate IgA-producing plasma cells, as well as differentiation of regulatory T cells (Tregs), thereby contributing to intestinal homeostasis. Short-chain fatty acid–producing bacteria further support TGF-β-mediated immunoregulation, establishing a dynamic circuit between microbiota, TGF-β, and host immunity. Together, these findings suggest a link between gut microbial activity, NAD^+^ availability, and TGF-β–mediated immune regulation.

Emerging evidence suggests that NAD^+^ metabolism is an important regulator of inflammation, fibrosis, and tissue homeostasis. NAD^+^ functions as a central metabolic coenzyme that plays essential roles in cellular redox reactions and serves as a substrate for key regulatory enzymes, including sirtuins, CD38, and poly (ADP-ribose) polymerases (PARPs) [28,29]. In mammalian cells, NAD ^+^ homeostasis is maintained through *de novo* synthesis from l-tryptophan, the Preiss–Handler pathway from nicotinic acid via nicotinate phosphoribosyltransferase (*Naprt*), and the salvage pathway, which recycles nicotinamide via nicotinamide phosphoribosyltransferase (*Nampt*) and nicotinamide mononucleotide adenylyltransferases (*Nmnats*). Beyond its intracellular roles, NAD^+^ metabolism contributes to systemic metabolic regulation and shapes host–microbiota interactions. For example, circulating nicotinamide can be utilized by gut microbiota to support microbial NAD^+^ biosynthesis in the colon [30]. Importantly, increasing intracellular NAD^+^ availability, either through metabolic enhancement or supplementation strategies, has been shown in preclinical models to alleviate tissue fibrosis and chronic inflammation across multiple organs [[31], [32], [33], [34], [35]].

In this study, we investigated how nicotinamide mononucleotide (NMN)-mediated NAD^+^ augmentation is associated with TGF-β–related transcriptional responses in mouse embryonic fibroblasts (MEFs) and murine models of colitis and colitis-associated cancer (CAC). We further examined the effects of NMN on inflammation, fibrosis, gut microbiota composition, and tumorigenesis using integrated transcriptomic profiling and microbiome analyses. Through these approaches, we sought to characterize transcriptional and microbial changes associated with NMN administration and to explore potential links among NAD^+^ metabolism, immune signaling, tissue remodeling, and host–microbiota interactions.

## 2 Materials and methods

### 2.1 Animals

Male and female C57BL/6J mice (5–7 weeks old) were purchased from CLEA Japan, Inc. (Tokyo, Japan) or The Jackson Laboratory (Bar Harbor, ME, USA). *Sirt1*^*flox/flox*^ mice (The Jackson Laboratory, #029603) were crossed with CAG-Cre transgenic mice (RIKEN BRC, #RBRC09807) to generate *Sirt1*^*ΔEx4/ΔEx4*^ mice. All mice were maintained under specific pathogen-free (SPF) conditions at 22 ± 2 °C under a 12-h light/dark cycle with ad libitum access to food and water. All animal procedures were approved by the Institutional Animal Care Committee of the Kindai University Faculty of Medicine (approval numbers: KAME-30-037, KAME-31-079, and KAME-2020-063). All experiments were performed in accordance with the institutional guidelines of Kindai University and applicable national regulations for the care and use of laboratory animals.

### 2.2 Study design

#### 2.2.1 Acute colitis model

Mice were randomly assigned to the experimental groups before DSS and/or NMN administration. Acute colitis was induced by administering 1.5% (w/v) dextran sulfate sodium (DSS; molecular weight 36,000–50,000 Da; MP Biomedicals, Canada) in the drinking water of 8-week-old mice for 7 consecutive days. The DSS solution was replaced every two days. Mice assigned to the NMN-treated and DSS + NMN-treated groups received 5 mM NMN (Oriental Yeast, Tokyo, Japan) in their drinking water for 7 days before DSS administration. Based on the monitored water intake (3–4.6 mL/day per mouse), the estimated NMN intake was approximately 15–22.8 μmol/day, corresponding to approximately 200–304 mg/kg/day assuming an average body weight of 25 g. Body weight was recorded daily. Stool consistency and rectal bleeding were monitored daily at the cage level. On Day 14, mice were euthanized, and the colon was excised. Tissues were snap-frozen and stored at −80 °C for molecular analyses or fixed in 10% neutral-buffered formalin for histological evaluation. Male mice were selected for the primary analyses because they are generally more susceptible to DSS-induced colitis than female mice, enabling robust and reproducible induction of colitis under standardized experimental conditions [36]. This decision was also supported by our preliminary observations of sex-dependent differences in body weight loss under identical DSS treatment conditions.

#### 2.2.2 Long-term administration of NMN

Mice were randomly assigned to the experimental groups before NMN administration. For long-term treatment, NMN (Oriental Yeast, Tokyo, Japan) was administered to 6-week-old male mice in the drinking water at a concentration of 5 mM for 15 weeks. NMN-containing water was replaced twice weekly to maintain NMN stability. Water intake was monitored throughout the experiment and ranged from 3.6 to 7.0 mL per mouse per day. This corresponded to an estimated daily NMN intake of approximately 18.1–35 μmol/day (approximately 240–470 mg/kg/day assuming an average body weight of 25 g).

#### 2.2.3 Colitis-associated cancer (CAC) model

Mice were randomly assigned to each group before AOM administration. The CAC model was established by intraperitoneal injection of azoxymethane (AOM; 12.5 mg/kg body weight; FUJIFILM Wako Pure Chemical Corporation, Osaka, Japan) into 5-week-old male and female mice on Day 0, followed by one week of regular drinking water. The mice were then administered 1.5% (w/v) DSS (MP Biomedicals, Canada) in their drinking water for 7 days, followed by 2 weeks of regular water. This DSS cycle was repeated three times. Mice assigned to the CAC + NMN group received 5 mM NMN (Oriental Yeast, Tokyo, Japan) in their drinking water continuously until the experimental endpoint. Based on the monitored water intake (2.5–7.1 mL/day per mouse), the estimated NMN intake was approximately 12.4–35.4 μmol/day (approximately 166–473 mg/kg/day assuming an average body weight of 25 g). At week 15 (approximately 100 days after AOM injection), mice were euthanized, and colonic tissues were harvested for further analyses. At the experimental endpoint, colons were opened longitudinally, and macroscopic tumors were counted under a stereomicroscope. The number of macroscopic tumors was quantified for each mouse.

### 2.3 Histology

#### 2.3.1 Hematoxylin and eosin (H&E) staining

Colon tissues were fixed in 10% neutral-buffered formalin (FUJIFILM Wako Pure Chemical Corporation, Osaka, Japan), embedded in paraffin, and sectioned at 10 μm. Sections were deparaffinized, rehydrated, and stained with hematoxylin and eosin. After dehydration through graded ethanol and clearing in G-NOX (GenoStaff, Tokyo, Japan), slides were mounted. Histological evaluation was performed on colon tissue sections obtained from a subset of mice in each experimental group. Histological inflammation was evaluated using the modified Dieleman scoring system by an investigator blinded to the treatment groups [37].

#### 2.3.2 Picrosirius red staining

Paraffin-embedded colon sections (10 μm) prepared as described above were deparaffinized, rehydrated, and stained using a Picrosirius Red Stain Kit (Polysciences, USA) according to the manufacturer's instructions. Briefly, sections were incubated in phosphomolybdic acid at room temperature for 2 min, followed by staining with picrosirius red solution for 90 min. Sections were then rinsed in acidified water for 2 min and 70% ethanol for 45 s. After dehydration through graded ethanol and clearing in G-NOX (GenoStaff, Tokyo, Japan), slides were mounted. Seven (control) and ten (DSS and DSS + NMN) non-overlapping fields from the distal colon were randomly selected at ×10 or ×20 magnification, depending on the size of the tissue section. Fibrotic areas were quantified using ImageJ and expressed as a percentage of the total tissue area. Image analysis was performed by an investigator blinded to the treatment groups.

### 2.4 Mouse embryonic fibroblast isolation and culture

Mouse embryonic fibroblasts (MEFs) were isolated from wild-type (WT) and *Sirt1*^*ΔEx4/ΔEx4*^ mouse embryos using a previously described protocol [31]. Briefly, embryos were collected at embryonic day 13.5, dissected free of extraembryonic tissues, and dissociated by mechanical disruption followed by incubation with trypsin–EDTA (FUJIFILM Wako Pure Chemical Corporation, Osaka, Japan). The resulting cell suspensions were cultured in Dulbecco's Modified Eagle Medium (DMEM; FUJIFILM Wako Pure Chemical Corporation, Osaka, Japan) supplemented with 10% fetal bovine serum (FBS), 100 U/mL penicillin, and 100 μg/mL streptomycin. MEFs between passages 4 and 6 were used for all experiments. Cells were maintained at 37 °C in a humidified incubator with 5% CO_2_. For RNA-seq and NAD^+^ assays, MEFs were cultured overnight in serum-free medium and stimulated with 5 ng/mL recombinant TGF-β1 (PeproTech, USA) for 24 h in the presence or absence of 0.1 mM NMN (Oriental Yeast Co., Ltd., Tokyo, Japan).

### 2.5 RNA isolation and bulk RNA sequencing

RNA-seq experiments were performed using independent biological replicates, with the number of replicates indicated in the corresponding figure legends. Sample sizes were as follows: MEFs (n = 2 per group), acute DSS colon (n = 3 per group), colon tissues from long-term NMN-treated mice (n = 5 per group), and CAC tumor tissues (n = 5 per group). Total RNA was extracted from cultured cells and colonic tissues using TRIzol™ Reagent (Thermo Fisher Scientific, USA). RNA purification was performed using either the Monarch® Total RNA Miniprep Kit (New England Biolabs, USA) or the RNeasy Mini Kit (Qiagen, Germany), depending on the sample type. RNA integrity and concentration were assessed using the Agilent 2100 Bioanalyzer (Agilent Technologies, USA). RNA-seq libraries were prepared using either the TruSeq RNA Sample Preparation Kit (Illumina, USA) or the NEBNext® Ultra™ Directional RNA Library Prep Kit for Illumina® (New England Biolabs, USA), following the respective manufacturer's protocols. Sequencing was performed on the Illumina HiSeq 2500 or NovaSeq 6000 platforms. Adapter trimming and quality filtering were performed using Trim Galore! (v0.6.10). Read quality was assessed using FastQC (v0.12.1) with default parameters.

Trimmed reads were aligned to the mouse reference genome (GRCm39) using HISAT2 (v2.2.1) with GENCODE vM34 annotation. Gene-level quantification was performed using the featureCounts function from the Subread package (v2.0.6). Additionally, transcript abundance (TPM) was calculated using StringTie (v2.2.1). For differential expression analysis, the resulting count matrix was imported into R and analyzed using the edgeR package (v4.6.3). Genes with logCPM > 2, FDR < 0.05 (Benjamini–Hochberg correction), and |log2FC| > 0.58 (corresponding to a 1.5-fold change) were considered differentially expressed.

Publicly available single-cell RNA sequencing data were obtained from the Broad Institute Single Cell Portal (study accession: SCP2038, “Single cell and spatial transcriptomics of mouse colon”). The single-cell expression matrix was processed using the Seurat package (v5.4.0) in R to generate cell type-specific reference signatures for CIBERSORTx. Cells were normalized, scaled, and clustered according to the standard Seurat workflow, and cell types were annotated based on canonical marker genes. Digital deconvolution analysis was performed using CIBERSORTx to estimate the relative abundance of cell populations in bulk RNA-seq samples. The resulting cell-type proportions were compared across experimental conditions.

Visualization was performed using the ggplot2 package (v3.5.2). Gene set enrichment analysis was conducted using the clusterProfiler package (v4.16.0), with multiple testing correction performed using the Benjamini–Hochberg method. The sequencing platforms and library preparation kits used for each cohort are summarized in Supplementary Table S1. To assess the reproducibility of RNA-seq datasets, principal component analysis (PCA), hierarchical clustering, and Pearson correlation analyses were performed using normalized expression values.

### 2.6 Bacterial DNA extraction from fecal samples and 16S rRNA gene sequencing

Fecal samples were collected from mice in the acute colitis model described in Section 2.2.1 (n = 4 per group) and stored at −80 °C until use. Bacterial genomic DNA was extracted using the QIAamp DNA Stool Mini Kit (Qiagen, Germany) according to the manufacturer's instructions. DNA concentration and purity were assessed using a NanoDrop spectrophotometer (Thermo Fisher Scientific). The V2–V9 hypervariable regions of the 16S rRNA gene were amplified using the Ion 16S™ Metagenomics Kit (Thermo Fisher Scientific). Libraries were prepared using the Ion Plus Fragment Library Kit with barcoded adapters, pooled, and sequenced on the Ion S5™ platform as single-end 400-bp reads. Raw FASTQ files were processed using QIIME2 (v2024.5), including quality filtering and denoising. Taxonomic assignment was primarily performed using the Genome Taxonomy Database (GTDB) to provide improved phylogenetic resolution. For comparison with conventional taxonomy frameworks, parallel analyses based on SILVA v138 were also conducted, yielding broadly similar trends despite minor taxonomic differences (Supplementary Fig. S6). Differential abundance analyses were performed in R using ANCOM-BC2 (v2.10.1), ALDEx2 (v1.40.0), and MaAsLin3 (v1.0.0) because these methods employ different statistical frameworks and assumptions for compositional microbiome data. The overlap among the three analytical approaches was evaluated to assess the consistency of the findings. Given the distinct statistical characteristics of these methods, the results were interpreted as exploratory observations rather than definitive consensus findings. Concordance of differential bacterial taxa identified by ANCOM-BC2, ALDEx2, and MaAsLin3 analyses is summarized in Supplementary Table S2. Functional profiles were predicted using PICRUSt2 (v2.5.3) and predicted pathway abundances were analyzed using DESeq2 (v1.50.2).

### 2.7 NAD assay

NAD^+^ levels were measured using a modified enzymatic cycling assay based on a previously described method [38]. NAD^+^ and NADH were extracted separately from MEFs using ice-cold 0.6 M perchloric acid and 0.25 N KOH in 50% ethanol, respectively. The lysates were diluted ten-fold with 100 mM phosphate buffer (pH 8.0) and kept on ice until use. NAD^+^ and NADH standards (FUJIFILM Wako Pure Chemical Corporation, Osaka, Japan) were included for quantification. 4 μL of each extract or standard was added to 100 μL of NAD^+^ cycling buffer, which contained 100 μg/mL bovine serum albumin (Sigma-Aldrich), 10 mM nicotinamide, 2% ethanol, 10 μM flavin mononucleotide, 20 μM resazurin, 100 μg/mL alcohol dehydrogenase, and 20 μg/mL diaphorase (all from FUJIFILM Wako Pure Chemical Corporation, Osaka, Japan) in 100 mM phosphate buffer (pH 8.0). Fluorescence intensity was measured at 590 nm with excitation at 540 nm using a 96-well black plate on an ARVO 1420 microplate reader (PerkinElmer, USA). Protein concentrations were determined using the BCA Protein Assay Kit (Thermo Fisher Scientific, USA).

### 2.8 Real-time mitochondrial respiration analysis

Real-time metabolic analysis was performed using the Seahorse XFe96 Analyzer (Agilent Technologies, USA) to assess mitochondrial respiration in mouse embryonic fibroblasts (MEFs) derived from wild-type (WT) and *Sirt1*^*ΔEx4/ΔEx4*^ mice. MEFs were seeded into Seahorse XF96 Cell Culture microplates at a density of 2 × 10^4^ cells/well and cultured overnight. Prior to the assay, the culture medium was replaced with Seahorse XF Base Medium (Agilent) supplemented with 10 mM glucose, 2 mM glutamine, and 1 mM pyruvate, and cells were incubated in a CO_2_-free incubator at 37 °C for 1 h. Mitochondrial respiration was evaluated using the Seahorse XF Cell Mito Stress Test (Agilent) according to the manufacturer's protocol. During the assay, oligomycin (1 μM), FCCP (1 μM), and a mixture of rotenone (0.5 μM) and antimycin A (0.5 μM) were sequentially injected. Oxygen consumption rate (OCR) was measured in real time and normalized to protein content. Data were analyzed using Wave software (Agilent Technologies).

### 2.9 Statistical analysis

All statistical analyses were performed using R (v4.5.1). Data are presented as mean ± SEM unless otherwise indicated. Sample sizes (n) for each analysis are provided in the corresponding figure legends. Statistical significance was defined as P < 0.05. Normality was assessed using the Shapiro–Wilk test, and statistical tests were selected accordingly. For comparisons between two groups, an unpaired two-tailed Student's t-test or Mann–Whitney *U* test was used as appropriate based on data distribution. For comparisons among three or more groups, one-way ANOVA followed by Tukey's multiple comparisons test was used for normally distributed data, whereas the Kruskal–Wallis test followed by Dunn's multiple comparisons test with Holm correction was used for non-parametric data. Histological inflammation scores (modified Dieleman score) were analyzed using the Kruskal–Wallis test followed by Dunn's multiple comparisons test with Holm correction. For RNA-seq data, differential expression analysis was performed using edgeR with Benjamini–Hochberg correction (FDR < 0.05). For microbiome analyses, differential abundance was assessed using ANCOM-BC2, ALDEx2, and MaAsLin3, with multiple-testing correction implemented according to each method. In regard with differential abundance analysis was performed using ALDEx2, statistical significance was assessed using the Benjamini–Hochberg-adjusted Wilcoxon rank-sum test (wi.eBH < 0.05), which was selected considering the small sample size and the non-parametric characteristics of microbiome abundance data.

## 3 Results

### 3.1 NMN alters TGF-β-induced gene expression in mouse embryonic fibroblasts

To characterize the transcriptional response to TGF-β, we performed RNA sequencing of mouse embryonic fibroblasts (MEFs) treated with TGF-β for 24 h. PCA and Pearson correlation analysis demonstrated reproducibility among biological replicates and clustering according to treatment conditions (Supplementary Fig. S1g and h). We identified 1153 upregulated and 1333 downregulated genes (|log_2_FC| > 0.58, logCPM > 2, FDR < 0.05). Consistent with previous reports, TGF-β treatment increased the expression of fibrosis-associated genes such as *Prg4*, *Timp1*, *Col7a1*, *Crispld2*, and *Tfpi2*, while decreasing the expression of the stem cell marker *Lgr5* (Supplementary Fig. S1a and b). Gene set enrichment analysis (GSEA) showed enrichment of the HIF-1 signaling pathway, Apoptosis, Inflammatory bowel disease, and TNF signaling pathway following TGF-β treatment (Supplementary Fig. S1c–f).

RNA-seq analysis identified transcriptional differences following NMN treatment in TGF-β-stimulated MEFs. In TGF-β-stimulated MEFs, 571 genes were upregulated and 278 genes were downregulated following NMN treatment (using the same criteria as described above). Importantly, genes associated with tissue remodeling rather than fibrotic deposition (*Adamts15*, *Efemp1*, and *Prelp*) showed increased expression following NMN treatment, suggesting differences in extracellular matrix-related gene expression.

**Fig. 1:**
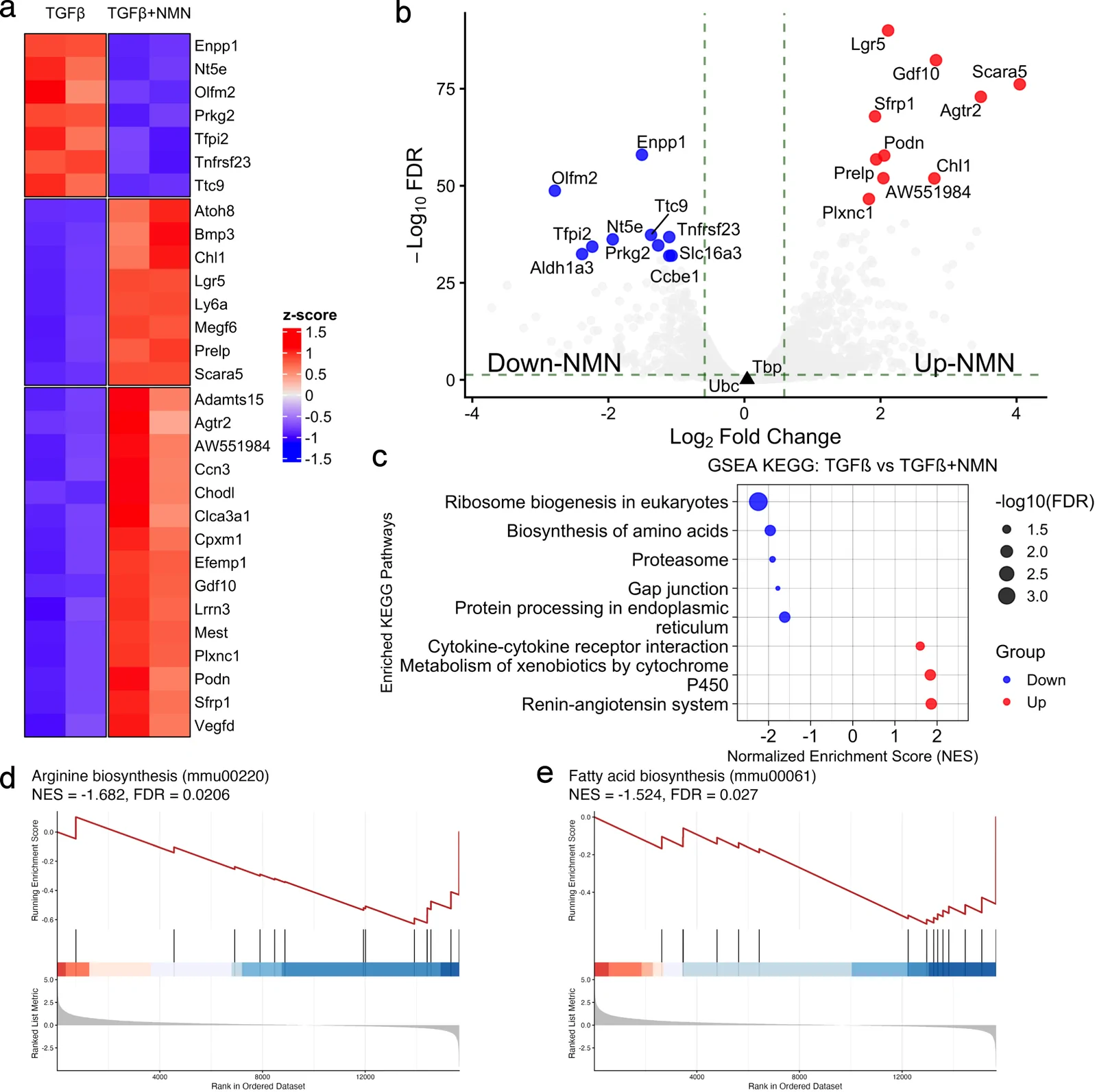
Gene expression profiles of TGF-β-treated MEFs following NMN treatment. RNA sequencing was performed using two independent biological replicates per group (n = 2). NMN treatment altered gene expression profiles in TGF-β-treated mouse embryonic fibroblasts (MEFs), with 571 upregulated and 278 downregulated genes (|log2FC| > 0.585, logCPM > 2, FDR < 0.05). (a) Heatmap of the top 30 differentially expressed genes. Gene expression values were scaled by row (z-score). Red and blue indicate relatively higher and lower expression levels, respectively. (b) Volcano plot showing differential gene expression between TGF-β-treated MEFs with and without NMN treatment. The top 10 upregulated and top 10 downregulated genes are highlighted in red and blue, respectively. (c) Dot plot of significantly enriched KEGG pathways identified by gene set enrichment analysis (GSEA) (NES > 1.5 or < −1.5, FDR < 0.05). (d, e) Representative GSEA enrichment plots of negatively enriched KEGG pathways, including Arginine biosynthesis (d) and Fatty acid biosynthesis (e).

Following NMN treatment, the expression of several genes repressed by TGF-β (e.g., *Lgr5*, *Gdf10*, and *Naprt*) was partially restored, while the expression of profibrotic genes such as *Tfpi2*, *Enpp1*, and *Nt5e* (CD73) was reduced (Fig. 1a and b). GSEA showed enrichment of the renin–angiotensin system, fatty acid biosynthesis, and arginine biosynthesis following NMN treatment (Fig. 1c–e). TGF-β stimulation was associated with changes in transcription factors and epigenetic regulators. Several genes, including *Twist1*, *Id1/2*, and DNA methylation–related genes (*Dnmt3a/3b*, *Tet1/2*), were downregulated, whereas *Twist2*, *Snai1/2*, and chromatin regulators such as *Smarca4* and *Eed* were upregulated. NMN treatment partially altered the expression of several of these factors (Supplementary Fig. S2b and c).

Intracellular NAD^+^ levels were reduced following TGF-β stimulation and increased following NMN treatment (Supplementary Fig. S2d). Consistent with these findings, mitochondrial respiratory function was increased in NMN-treated MEFs, suggesting a potential association with NAD^+^-dependent pathways (Supplementary Fig. S2e and f). These findings suggest an association between NAD ^+^ metabolism and mitochondrial respiratory function following NMN treatment. Expression of the NMN transporter *Slc12a8* was not significantly altered by TGF-β but showed a modest increase upon NMN treatment (Supplementary Fig. S2a). *Naprt* expression was decreased following TGF-β stimulation, whereas its expression was increased following NMN treatment. In addition, genes involved in NAD^+^ metabolism were differentially regulated, with *Nmnat2* and *Sirt4* downregulated and *Nampt* (*Pbef*) and mitochondrial *Nmnat3* upregulated by TGF-β. Other NAD^+^-related genes remained largely unchanged. These findings suggest that TGF-β stimulation is associated with changes in NAD^+^ metabolism, which may be related to the observed transcriptional changes.

### 3.2 NMN administration was associated with altered fibrosis- and inflammation-related gene expression profiles in DSS-induced colitis

To investigate the effects of NMN on fibrosis and inflammation *in vivo*, we analyzed colonic tissues from a dextran sulfate sodium (DSS)-induced IBD mouse model, originally established by *Okayasu* et al. [39]. Male mice were used for subsequent analyses because preliminary experiments demonstrated lower susceptibility of female mice to DSS-induced colitis under the same experimental conditions. NMN administration partially attenuated DSS-induced reductions in colon length and body weight (Supplementary Fig. S3a–c). In mice receiving NMN alone for 2 weeks, colon length and body weight did not differ from those of control mice (Supplementary Fig. S4). Histological analysis showed that NMN administration was associated with reduced fibrosis in the colon compared with DSS-treated mice, whereas histological inflammation scores assessed by the modified Dieleman score were not significantly different between groups (Supplementary Fig. S3d–k). PCA and Pearson correlation analysis demonstrated reproducibility among biological replicates and clustering according to treatment conditions (Supplementary Fig. S5a and b). Gene expression patterns in this model were broadly consistent with those reported in human IBD patients and other experimental IBD models. Analysis of significantly altered genes revealed enrichment of pathways related to TGF-β signaling (Supplementary Fig. S5c–h).

**Fig. 2:**
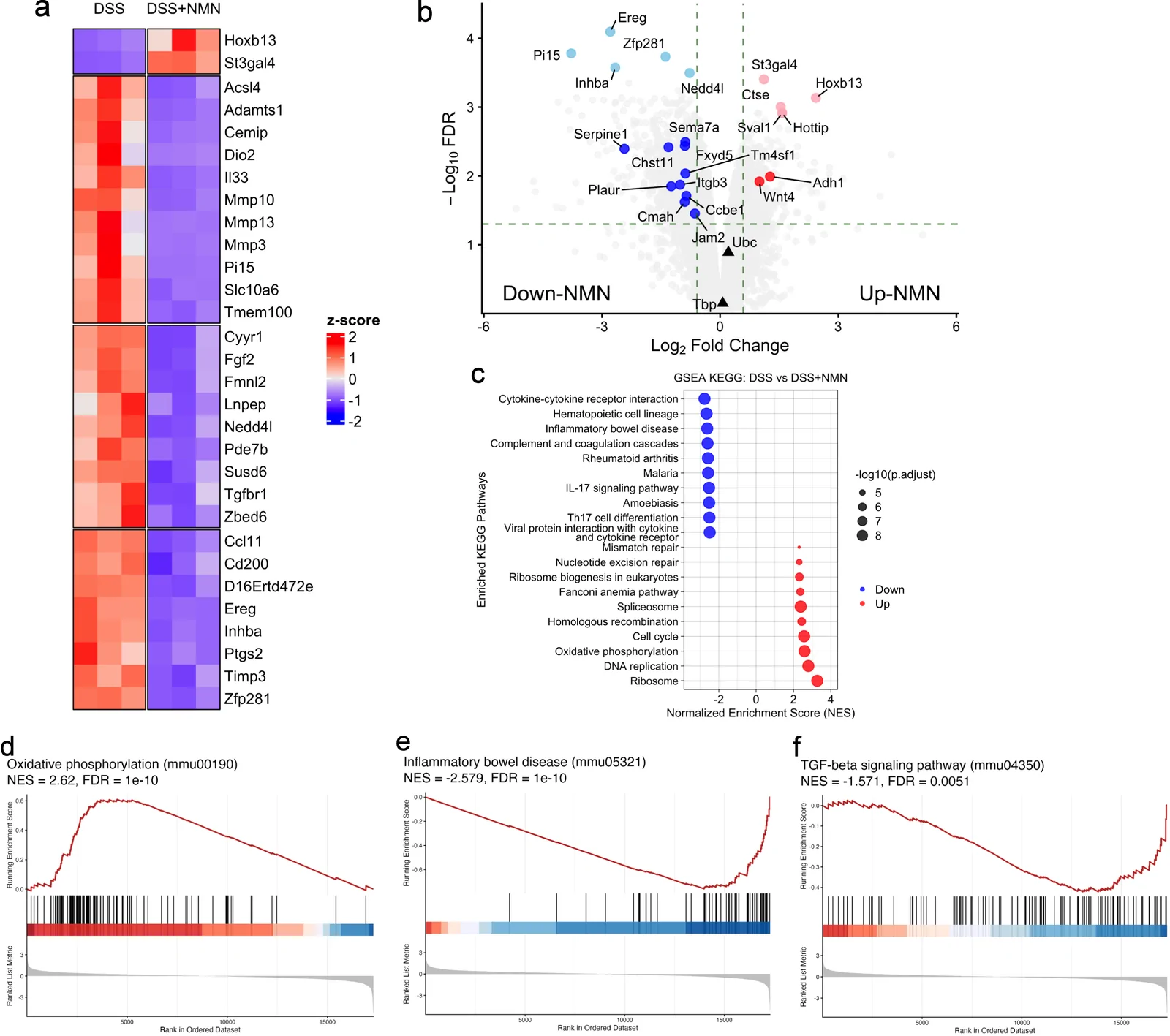
Gene expression changes associated with NMN administration in the colon of DSS-treated mice. RNA sequencing was performed using three independent biological replicates per group (n = 3). NMN administration altered gene expression profiles in the colon of DSS-treated mice, with 451 upregulated and 878 downregulated genes (|log2FC| > 0.585, logCPM > 2, FDR < 0.05). (a) Heatmap of the top 30 differentially expressed genes. Gene expression values were scaled by row (z-score). Red and blue indicate relatively higher and lower expression levels, respectively. (b) Volcano plot showing differential gene expression between DSS-treated mice and DSS-treated mice receiving NMN. The top 5 upregulated and top 5 downregulated genes are highlighted in red and blue, respectively. Genes showing consistent NMN-associated expression changes following NMN administration in both TGF-β-treated MEFs and DSS-treated mice are indicated separately, with upregulated genes shown in light red and downregulated genes in sky blue. (c) Dot plot of significantly enriched KEGG pathways identified by gene set enrichment analysis (GSEA) (FDR < 0.05). (d–f) Representative GSEA enrichment plots of KEGG pathways associated with NMN administration in DSS-treated mice. Oxidative phosphorylation showed positive enrichment (d), whereas Inflammatory bowel disease (e) and TGF-beta signaling pathway (f) showed negative enrichment in NMN-treated samples.

Transcriptomic profiling of colonic tissues in the DSS-induced IBD mouse model identified differential gene expression between NMN-treated and DSS-only groups, with 451 genes upregulated and 878 genes downregulated (|log_2_FC| > 0.58, logCPM > 2, FDR < 0.05). Upregulated genes included *Hoxb13* and *St3gal4*, whereas genes associated with fibrosis and inflammation, including *Inhba*, *Mmp3*, and *Ptgs2*, showed decreased expression (Fig. 2a and b). Notably, *Adh1* and *Wnt4* showed increased expression following NMN administration in colon tissues of the IBD mouse model. Similar expression changes were observed in TGF-β-stimulated MEFs following NMN treatment. Conversely, NMN administration consistently downregulated *Inhba*, *Serpine1*, *Plaur*, *Sema7a*, and *Chst11* in the colon tissues, which have been implicated in fibroblast activation, extracellular matrix remodeling, and inflammatory signaling (Fig. 2b).

KEGG-based GSEA of colon tissues in the IBD mouse model identified enrichment of oxidative phosphorylation and several other pathways in NMN-treated mice, whereas pathways associated with inflammatory bowel disease, Th17 cell differentiation, and TGF-β signaling showed reduced enrichment (Fig. 2c–f). These findings suggest that NMN administration is associated with changes in fibrosis-, inflammation-, and metabolism-related transcriptional profiles in DSS-induced colitis.

### 3.3 NMN administration alters the fecal microbiota in a DSS-induced colitis model

**Fig. 3:**
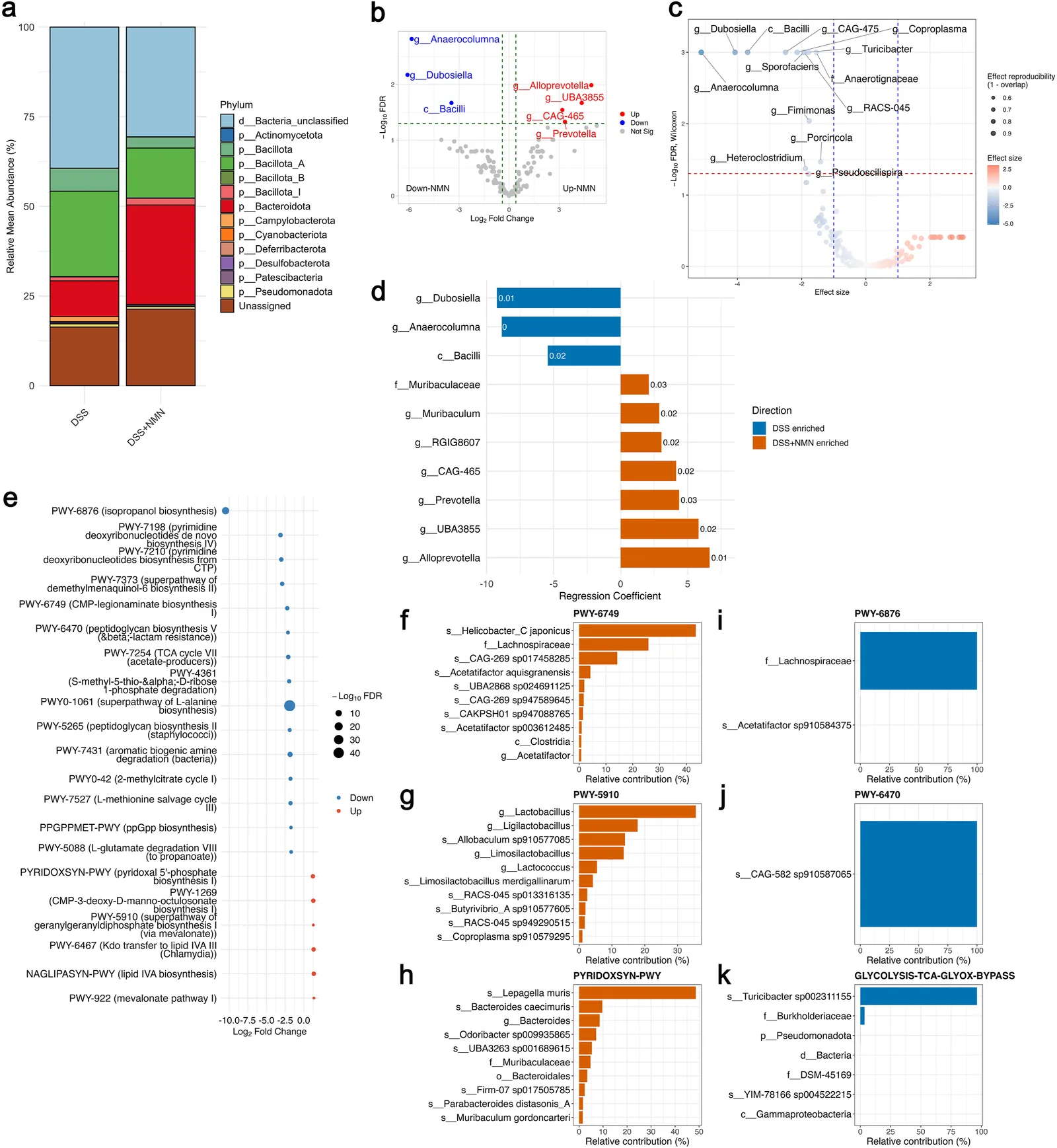
Gut microbiota composition and predicted microbial functions in DSS-treated mice with or without NMN administration. 16S rRNA gene sequencing was performed using four independent biological replicates per group (n = 4). Fecal samples from DSS-treated mice with or without NMN treatment were analyzed by 16S rRNA gene sequencing. (a) Stacked bar plot showing the relative abundance of bacterial phyla (mean across samples). (b) Volcano plot of differential genus abundance based on ANCOM-BC2 analysis. Genera significantly increased in NMN-treated mice are shown in red, and those decreased are shown in blue (|log2FC| > 0.585, FDR < 0.05). (c) Bubble plot of differentially abundant genera identified by ALDEx2. The x-axis indicates effect size, and the y-axis indicates −log10 (Wilcoxon FDR, wi.eBH). Bubble size represents effect reproducibility, calculated as 1 − overlap, and color indicates effect size. Dashed lines denote thresholds of |effect size| = 1 and FDR = 0.05. (d) Genus-level differential abundance analysis using MaAsLin3 (GTDB r220). Bars represent regression coefficients; negative coefficients indicate higher abundance in DSS mice, whereas positive coefficients indicate higher abundance in DSS + NMN mice (FDR < 0.05). (e) Bar plot of significantly altered MetaCyc pathways predicted by PICRUSt2. (f–h) Contributions of individual taxa to selected MetaCyc pathways showing positive enrichment (PWY-6749, PWY-5910, and PYRIDOXSYN-PWY). (i–k) Contributions of individual taxa to selected MetaCyc pathways showing negative enrichment (PWY-6876, PWY-6470, and GLYCOLYSIS-TCA-GLYOX-BYPASS).

To characterize the fecal microbiota following NMN administration, we performed 16S rRNA gene sequencing in DSS-induced colitis mice. NMN-treated mice showed shifts in the relative abundance of major bacterial phyla, including *Bacillota* and *Bacteroidota*, as classified by the GTDB reference database (Fig. 3a). Differential abundance analyses identified taxa showing significant differences following NMN administration. At the genus level, NMN-treated mice showed increased relative abundances of *Alloprevotella*, *Prevotella*, and *RGIG8607* (family Lachnospiraceae), as well as an uncultured taxon (*UBA3855*). Some of these taxa have previously been linked to microbial metabolic functions, including carbohydrate utilization and short-chain fatty acid-related metabolism. In contrast, *Anaerocolumna* and *Dubosiella* showed decreased relative abundances in NMN-treated mice (Fig. 3b–d). Differential abundance analyses using multiple approaches identified overlapping microbial taxa. Four increased taxa were consistently detected by ANCOM-BC2 and MaAsLin3, whereas three decreased taxa were identified across all three methods (ANCOM-BC2, ALDEx2, and MaAsLin3), suggesting similar directional trends among statistical approaches (Supplementary Table S2). Several taxa, including *Anaerocolumna* and *Dubosiella* (decreased) and *Alloprevotella*, *UBA3855*, and *CAG-465* (increased), showed consistent directional changes across multiple methods. These findings suggest reproducible patterns of NMN-associated microbiota shifts, although the results remain exploratory given the limited sample size.

Functional prediction using PICRUSt2 identified higher predicted abundances of pathways related to membrane biosynthesis, isoprenoid metabolism, and vitamin B6 biosynthesis (Fig. 3e). In contrast, pathways associated with antimicrobial resistance, stress responses, and alternative carbon utilization were less represented in NMN-treated mice. Although the PICRUSt2 outputs are inferred rather than directly measured, contribution analysis indicated that the predicted functional profiles were associated with multiple taxa, including SCFA-related genera and inflammation-associated bacteria (Fig. 3f–k). Alpha and beta diversity were not significantly different between groups, suggesting that the observed microbial changes were primarily reflected at the level of specific taxa rather than overall community diversity (Supplementary Fig. S6d and e).

### 3.4 Long-term NMN administration alters metabolic and immune gene expression in normal colon and colitis-associated tumors

**Fig. 4:**
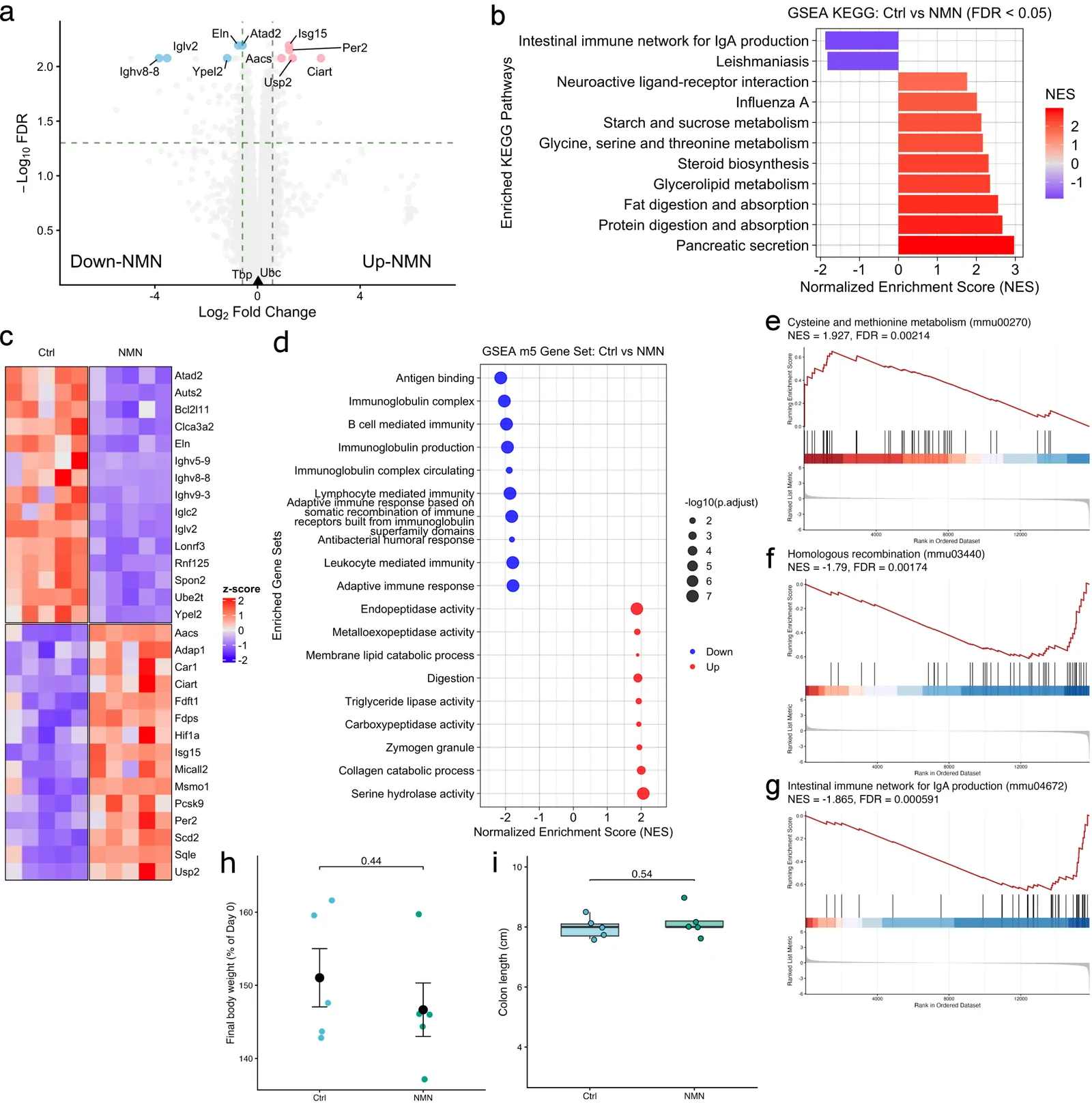
Gene expression profiles in the colon of C57BL/6J mice following long-term NMN administration. RNA sequencing was performed using five independent biological replicates per group (n = 5). Comparison of colonic gene expression between untreated control mice and mice treated with NMN for 100 days identified 51 upregulated and 66 downregulated genes (|log2FC| > 0.585, logCPM > 2, FDR < 0.05). (a) Volcano plot of differential gene expression. The top 5 upregulated and top 5 downregulated genes are highlighted in light red and sky blue, respectively. (b) Bar plot of significantly enriched KEGG pathways identified by gene set enrichment analysis (GSEA) (FDR < 0.05). (c) Heatmap of the top 30 differentially expressed genes. Gene expression values were scaled by row (z-score). Red and blue indicate relatively higher and lower expression levels, respectively. (d) Dot plot of Gene Ontology (GO) enrichment based on GSEA (FDR < 0.05). (e–g) Representative GSEA enrichment plots of KEGG pathways associated with long-term NMN administration, including Cysteine and methionine metabolism (e), Homologous recombination (f), and Intestinal immune network for IgA production (g). (h) Final body weight expressed as a percentage of the initial body weight (Day 0) (untreated control, n = 5; NMN, n = 5). (i) Quantification of colon length (untreated control, n = 5; NMN, n = 5). Data are presented as mean ± SEM. Statistical significance was determined using an unpaired Student's t-test.

To examine the effects of long-term NMN administration, we performed RNA sequencing of colonic tissues from wild-type C57BL/6J mice following approximately 100 days of NMN administration. In mice receiving NMN alone for 100 days, colon length and body weight did not differ from those of control mice (Fig. 4h and i). Compared with control mice, 51 genes were upregulated and 66 genes were downregulated following NMN administration (|log_2_FC| > 0.58, logCPM > 2, FDR < 0.05). PCA and Pearson correlation analysis demonstrated reproducibility among biological replicates and clustering according to treatment conditions (Supplementary Fig. S7). Upregulated genes included those associated with circadian regulation and metabolic processes, such as *Ciart*, *Usp2*, *Per2*, *Hif1a,* and *Hmgcs1*, whereas genes associated with extracellular matrix organization and fibrosis, including *Eln* and *Spon2*, were downregulated (Fig. 4a–c). GSEA identified enrichment of metabolic pathways, including steroid biosynthesis and amino acid metabolism, whereas immune-related pathways, such as the intestinal immune network for IgA production and lymphocyte-mediated immunity, showed reduced enrichment (Fig. 4b–d–g).

To determine whether NMN influences tumorigenesis, we next analyzed a colitis-associated cancer (CAC) model induced by azoxymethane (AOM) and dextran sulfate sodium (DSS) over a similar time frame. Tumors developed in both control and NMN-treated groups, indicating that NMN administration did not significantly affect tumorigenesis (Supplementary Fig. S8). Similar observations were obtained in female mice. Although female mice developed fewer tumors than males, NMN did not significantly alter tumor number (Supplementary Fig. S12). RNA sequencing of tumor tissues from male mice identified only a limited number of differentially expressed genes in NMN-treated mice, including 5 upregulated and 10 downregulated genes under the same criteria (Supplementary Fig. S10a and b). PCA and Pearson correlation analysis demonstrated reproducibility among biological replicates and clustering according to treatment conditions (Supplementary Fig. S9).

Among the downregulated genes were *Ephx2* and the *lncRNA H19*, both previously associated with inflammation and tumor progression. Notably, several genes showing reduced expression in NMN-treated tumors, including *Tsc22d1*, *Peg3*, and *H19*, have previously been associated with TGF-β signaling, apoptosis, epithelial–mesenchymal transition, and fibrosis. Consistent with these gene-level changes, GSEA identified enrichment of gene sets related to antigen processing and presentation and lymphocyte-mediated immunity in NMN-treated tumors (Supplementary Fig. S10d–g). In addition, digital deconvolution analysis suggested a reduction in mast cell signatures, although these cells were present at low abundance in control tumors and the biological significance of this finding remains unclear (Supplementary Fig. S10c).

To provide context for these NMN-associated changes, we characterized the baseline transcriptional landscape of the DSS-induced CAC model. Tumor tissues showed increased expression of Wnt signaling- and stemness-associated genes, together with inflammatory and extracellular matrix–related genes (Supplementary Fig. S11).

## 4 Discussion

This study provides an integrated transcriptomic and microbiome-level analysis of the effects of NMN administration under TGF-β–associated inflammatory conditions. By integrating transcriptomic analyses in MEFs with *in vivo* studies in IBD and colitis-associated cancer models, we characterized transcriptional changes associated with NMN administration, including changes in genes related to NAD^+^ metabolism.

In MEFs, NMN altered the expression of numerous TGF-β-responsive genes, many of which have previously been associated with fibrosis and inflammation, supporting an interaction between NAD^+^ metabolism and TGF-β-associated transcriptional programs. TGF-β stimulation also altered the expression of several genes involved in NAD^+^ metabolism, with increased expression of *Nampt* and *Nmnat3* and reduced expression of *Naprt*, *Nmnat2*, and *Sirt4* (Supplementary Fig. S2a). NAPRT and NAMPT are the rate-limiting enzymes of the Preiss–Handler and NAD^+^ salvage pathways, respectively. Their reciprocal expression changes suggest that TGF-β remodels the transcriptional program governing NAD^+^ biosynthetic pathways, although the functional contribution of the Preiss–Handler pathway under standard culture conditions lacking nicotinic acid is likely limited. Despite increased *Nampt* expression, intracellular NAD^+^ levels were reduced, suggesting that compensatory induction of *Nampt* was insufficient to offset increased NAD ^+^ consumption or other metabolic demands induced by TGF-β (Supplementary Fig. S2d). NMN partially restored *Naprt* expression without substantially affecting *Nampt*, suggesting possible remodeling of NAD^+^ biosynthetic pathways. However, this hypothesis requires validation by metabolomic or isotope-tracing approaches.

Beyond changes in NAD^+^ metabolism–related genes, GSEA identified enrichment of several metabolic pathways, including the renin–angiotensin system, fatty acid biosynthesis, and arginine biosynthesis, following NMN administration (Fig. 1c–e; Supplementary Fig. S1c–f). Although these pathway-level associations are exploratory, they suggest that NMN-associated transcriptional changes extend beyond fibrosis- and inflammation-related genes to broader metabolic programs. In our MEF model, 24 h TGF-β stimulation may represent an early phase of the transcriptional response, although additional time-course studies will be required to determine its reversibility. Consistent with this, *Tet1/2* and *Dnmt3a/3b* showed reduced expression after 24 h of TGF-β stimulation (Supplementary Fig. S2c). In addition, *Twist1* expression was reduced, whereas *Twist2* expression was increased, indicating distinct regulation of these transcription factors during the TGF-β response in MEFs (Supplementary Fig. S2b).

In our DSS-induced colitis model, NMN altered the expression of genes related to epithelial function, fibrosis, and inflammation, consistent with reduced TGF-β-associated tissue remodeling. Several genes that responded to both TGF-β and NMN in MEFs, including *Adh1* and *Wnt4*, also showed increased expression in NMN-treated colonic tissues, whereas fibrosis- and inflammation-associated genes, including *Inhba*, *Serpine1*, *Plaur*, *Sema7a*, and *Chst11*, showed reduced expression (Fig. 2b). Consistent with these gene-level changes, GSEA identified reduced enrichment of inflammatory bowel disease, Th17 cell differentiation, and TGF-β signaling pathways (Fig. 2c–f). Although these observations are based on transcriptomic analyses and do not establish direct regulation of TGF-β signaling, they are consistent with coordinated transcriptional changes associated with TGF-β-related tissue remodeling.

SIRT1 has been reported to regulate TGF-β signaling through interactions with SMAD family proteins, including SMAD3, although its precise role remains controversial [40]. In several fibrotic disorders, TGF-β signaling is enhanced whereas SIRT1 expression is reduced [41]. However, the functional consequences of SIRT1 activation appear to be context dependent. For example, SIRT1 activation has been reported to enhance TGF-β signaling and collagen production in systemic sclerosis, whereas other studies have shown that SIRT1 attenuates TGF-β–dependent transcription through deacetylation of SMAD3, thereby suppressing fibrotic responses [42]. These findings suggest that the interaction between SIRT1 and SMAD signaling is likely to depend on cell type and disease context. Future studies using colon-specific *Sirt1* knockout mice will be important to determine whether SIRT1-dependent regulation of SMAD acetylation contributes to intestinal inflammation, fibrosis, and colitis-associated tumorigenesis.

Recent studies suggest that gut microbes can utilize host-derived nicotinamide as a source for NAD^+^ biosynthesis, and supplementation with NAD^+^ precursors such as NMN may influence microbial metabolic activity [30,43]. In our DSS-induced colitis model, NMN was associated with changes in the relative abundance of several genera, including *Alloprevotella*, a genus previously reported to vary under different dietary and inflammatory conditions [44]. However, whether these microbial alterations contribute to the effects of NMN remains unclear. Concordance analysis using three complementary differential abundance methods (ANCOM-BC2, ALDEx2, and MaAsLin3) identified several taxa showing consistent directional trends; however, these findings should be interpreted cautiously because of the limited sample size and the exploratory nature of microbiome analyses. PICRUSt2-based functional prediction suggested enrichment of pathways related to isoprenoid biosynthesis and pyridoxal 5′-phosphate (PLP) biosynthesis following NMN administration. These predicted functional changes may be relevant to microbial metabolism and host immune function, and reduced circulating PLP levels have been reported in patients with inflammatory bowel disease [45]. Conversely, NMN administration was associated with reduced predicted abundance of pathways related to antimicrobial resistance, stress responses (e.g., ppGpp biosynthesis), and alternative energy-generating pathways such as the glyoxylate bypass. Although these findings are based on inferred functional profiles, they suggest that NMN may be associated with alterations in host–microbe interactions through changes in predicted microbial metabolic potential. However, whether these microbiota alterations directly contribute to the biological effects of NMN remains unclear. Future studies using microbiota-targeted approaches, including fecal microbiota transplantation or germ-free models, will be required to distinguish microbiota-dependent effects from direct host responses to NMN.

As discussed above, maintaining NAD^+^ metabolism may contribute to tissue disease resilience. Long-term NMN administration has been shown to be safe in humans and to improve insulin sensitivity and age-related muscle dysfunction [[46], [47], [48]]. In our mouse model, long-term NMN administration increased expression of circadian rhythm–related genes, including *Per2*, *Ciart*, *Dbp*, *Hlf* and *Tef*. These genes have been reported to change during aging [[49], [50], [51]]. At the same time, immunoglobulin genes (*Igkc*, *Igha*, *Ighv3*-*8*) and inflammation-related genes (*Ly6c2*) were altered, consistent with changes in immune-related transcriptional programs [52]. However, because fecal or mucosal IgA levels were not measured, the functional consequences of altered immunoglobulin gene expression remain unclear. Future studies incorporating functional assessments of IgA secretion, such as measurements of fecal or mucosal IgA, will be necessary to determine whether these transcriptional changes influence mucosal immune homeostasis. Moreover, increased expression of lipid metabolism–related genes (*Sqle*, *Hmgcs1*) may support epithelial homeostasis and metabolic adaptation [53]. Collectively, these findings suggest that long-term NMN administration is associated with coordinated transcriptional changes in circadian regulation, immune-related pathways, and metabolism in the colon.

In the AOM/DSS-induced colitis-associated cancer (CAC) model, long-term NMN administration did not alter tumor number in either male or female mice. TGF-β is well recognized as having a dual role in colorectal tumorigenesis, acting as a tumor suppressor during early stages of tumorigenesis while promoting tumor progression in established cancers. Although NMN was associated with reduced fibrosis and altered TGF-β-associated transcriptional programs, these changes were not sufficient to reduce tumor development in our model. These findings suggest that modulation of fibrosis- and TGF-β-associated transcriptional programs alone may not be sufficient to influence tumor initiation under these experimental conditions. In addition, the number of differentially expressed genes identified in CAC tissues was limited, which may have restricted our ability to detect broader transcriptional changes associated with NMN treatment. Therefore, the effects of NMN on tumor-associated molecular pathways in this model should be interpreted with caution. Further studies will be important to clarify how NAD^+^ metabolism interacts with fibrosis, inflammation, and TGF-β signaling during different stages of colorectal tumorigenesis.

Peptide-based therapies, including glucagon-like peptide-1 (GLP-1) receptor agonists, have recently emerged as promising therapeutic approaches for IBD [54]. Whether NMN supplementation may provide complementary benefits in combination with these therapies remains speculative and warrants future investigation. In the present study, NMN was administered prophylactically prior to DSS exposure. This experimental design represents a preventive intervention model rather than a therapeutic treatment model. Although our findings suggest potential beneficial effects of prophylactic NMN administration, further studies evaluating therapeutic administration after disease onset will be required to determine its therapeutic potential and translational relevance. The NMN dose used in the present study exceeds doses typically evaluated in human clinical trials. Therefore, future studies should determine the dose–response relationship of NMN and evaluate whether lower doses produce comparable biological effects in order to better assess its clinical translatability. In addition, male mice were used in the primary analyses because they exhibited greater susceptibility to DSS-induced colitis under our experimental conditions. Consistent with previous reports and our preliminary observations, female mice showed lower susceptibility to DSS-induced colitis [36,55]. Future studies directly comparing male and female mice will be important to evaluate potential sex-dependent responses to NMN. Furthermore, although RNA-seq analyses across multiple experimental models provided comprehensive transcriptional profiles, the selected differentially expressed genes were not independently validated by qPCR or protein-level assays. In addition, direct measurements of colonic NAD^+^/NADH levels were not performed *in vivo*, and therefore the extent to which NMN supplementation altered tissue NAD^+^ metabolism under the experimental conditions remains to be determined. Future studies will be required to confirm these transcriptomic findings and clarify their biological significance. Finally, microbiome analyses in the present study were based on 16S rRNA sequencing and PICRUSt2-based functional prediction; therefore, direct measurements of microbial metabolites and functional validation will be required to determine the biological significance of these microbiota-associated changes.

In summary, our study demonstrated that NMN supplementation, which increases NAD^+^ availability, was associated with changes in gene expression programs related to metabolism, fibrosis, inflammation, and host–microbiome interactions. These findings support the concept that alterations in NAD^+^ metabolism are associated with coordinated transcriptional changes linked to chronic inflammation, fibrotic remodeling, and tissue homeostasis. Although additional studies, including human clinical investigations, are required, our findings provide a basis for further investigating the role of NAD^+^ metabolism in IBD, fibrosis-related diseases, and age-associated physiological decline. Together, our transcriptomic and microbiome analyses provide a resource for future mechanistic and translational studies of NAD^+^ metabolism.

## Author contributions

H.A. conceptualized the study, performed experiments, analyzed the data, wrote the original draft, and acquired funding. H.O. supervised the project and edited the manuscript. All authors provided resources, validated the data, and approved the final version of the manuscript.

## Ethics declaration

This study was conducted in accordance with the following guidelines for animal welfare and/or reporting: The institutional guidelines of Kindai University. This study was approved by The Animal Care and Use Committee of Kindai University. (Approval No. KAME-30-037, KAME-31-079, and KAME-2020-063)

## Data availability statement

### Materials availability

Materials used in this study, including genetically modified mice and cell lines, were obtained from commercial vendors (e.g., The Jackson Laboratory and CLEA Japan). No new reagents, genetically modified organisms, or cell lines were generated in this study. All other reagents and materials used are commercially available.

### Data and code availability

#### Raw data

All sequencing datasets generated in this study have been deposited in the DDBJ Sequence Read Archive (DRA) and are available under the following BioProject accession numbers:

• Bulk RNA-seq datasets:
  • MEF bulk RNA-seq (TGF-β/NMN treatment): PRJDB39655
  • Mouse colon bulk RNA-seq (short-term DSS/NMN experiments): PRJDB39657
  • Mouse colon bulk RNA-seq (long-term NMN administration): PRJDB39658
  • AOM/DSS-induced colon tumor RNA-seq: PRJDB39659
• 16S rRNA amplicon sequencing dataset:
  • Mouse fecal 16S rRNA sequencing (DSS-induced IBD model with NMN administration): PRJDB39660

Code availability: All analyses were performed using publicly available software packages as described in the Methods section. The analysis scripts used in this study are available from the corresponding author upon reasonable request.

Additional information: Any additional information required to reanalyze the data reported in this paper is available from the corresponding author upon request.

## Declaration of competing interests

The authors declare that they have no competing interests.

## Appendix A Supplementary data

Fig. S1 Gene expression profiles of TGF-β-treated MEFs. RNA sequencing was performed using two independent biological replicates per group (n = 2). TGF-β treatment of mouse embryonic fibroblasts (MEFs) resulted in 1153 upregulated and 1333 downregulated genes (|log2FC| > 0.585, logCPM > 2, FDR < 0.05). (a) Heatmap of the top 30 differentially expressed genes. Gene expression values were scaled by row (z-score). Red and blue indicate relatively higher and lower expression levels, respectively. (b) Volcano plot showing differential gene expression between TGF-β-treated and untreated MEFs. The top 10 upregulated and top 10 downregulated genes are highlighted in red and blue, respectively. (c) Dot plot of significantly enriched KEGG pathways identified by gene set enrichment analysis (GSEA) (FDR < 0.05). (d–f) Representative GSEA enrichment plots of negatively enriched KEGG pathways, including Apoptosis (d), Inflammatory bowel disease (e), and TNF signaling pathway (f). (g) Quality assessment of RNA-seq samples by principal component analysis (PCA). PCA was performed using normalized logCPM values to visualize the overall similarity and separation among untreated control MEFs, TGF-β-treated MEFs, and NMN-treated TGF-β-treated MEFs. (h) Correlation heatmap showing Pearson correlation coefficients among all RNA-seq samples.

Fig. S2 Effects of TGF-β and NMN on gene expression and metabolism in MEFs. (a–c) RNA-seq analysis showing expression in MEFs treated with TGF-β and/or NMN. Bar plots show expression levels (normalized counts) of (a) 13 genes involved in NAD^+^ metabolism, (b) 15 secondary response genes associated with TGF-β treatment, and (c) 24 genes associated with epigenetic memory. (d) Intracellular NAD^+^ levels measured by an NAD^+^ assay. TGF-β treatment for 24 h was associated with reduced NAD^+^ levels, whereas co-treatment with NMN partially restored NAD^+^ levels. (e) Real-time metabolic flux analysis of wild-type and *Sirt1*-deficient MEFs with or without NMN. (f) Bar plots of oxygen consumption rates (OCR) parameters, including basal, ATP-linked, maximal, and spare respiratory capacities, in wild-type and *Sirt1*-deficient MEFs.

Fig. S3 Histological analysis of DSS-induced colitis in mice. (a) Representative gross images of the colon and spleen from untreated control mice, DSS-treated mice, and DSS-treated mice receiving NMN. (b) Quantification of colon length (control, n = 11; DSS, n = 10; DSS + NMN, n = 12). (c) Body weight changes during the experimental period (control, n = 11; DSS, n = 10; DSS + NMN, n = 12). (d–f) Representative hematoxylin and eosin (H&E)-stained colon sections from untreated control, DSS-treated, and DSS + NMN mice. (g) Quantification of histological inflammation scores. (control, n = 4; DSS, n = 4; DSS + NMN, n = 3). (h–j) Representative Picrosirius red-stained colon sections showing collagen deposition in untreated control, DSS-treated, and DSS + NMN mice. (k) Quantification of fibrotic areas in the Picrosirius red-stained sections using ImageJ (control, n = 7; DSS, n = 10; DSS + NMN, n = 10). Data are presented as mean ± SEM. Colon length, body weight, and fibrotic area were analyzed using one-way ANOVA followed by Tukey's multiple comparisons test. Histological scores were analyzed using the Kruskal–Wallis test followed by Dunn's multiple comparisons test.

Fig. S4 No adverse effects of NMN administration in healthy mice. (a) Representative gross images of the colon and spleen from untreated control mice and NMN-treated mice. (b) Quantification of colon length (untreated control, n = 2; NMN, n = 3). (c) Body weight changes during the 2-week treatment period (untreated control, n = 2; NMN, n = 3). Data are presented as mean ± SEM. Statistical analyses were performed using an unpaired Student's t-test.

Fig. S5 Gene expression profiles in the acute DSS-induced colitis model. RNA sequencing was performed using three independent biological replicates per group (n = 3). Compared with untreated control mice, DSS administration for 7 days resulted in 1772 upregulated and 1021 downregulated genes (|log2FC| > 0.585, logCPM > 2, FDR < 0.05). (a) Quality assessment of RNA-seq samples by principal component analysis (PCA). PCA was performed using normalized logCPM values from all four experimental groups (untreated control, NMN-treated, DSS-treated, and DSS + NMN-treated mice). (b) Correlation heatmap showing Pearson correlation coefficients among all RNA-seq samples. (c) Heatmap of the top 30 differentially expressed genes. Gene expression values were scaled by row (z-score). Red and blue indicate relatively higher and lower expression levels, respectively. (d) Volcano plot showing differential gene expression between untreated control and DSS-treated mice. The top 5 upregulated and top 5 downregulated genes are shown in light red and sky blue, respectively. Genes showing consistent expression changes in both TGF-β-treated MEFs and DSS-treated mice are highlighted separately, with commonly upregulated genes shown in red and commonly downregulated genes shown in blue. (e, f) Representative GSEA enrichment plots of KEGG pathways associated with DSS treatment, including Inflammatory bowel disease (e) and Oxidative phosphorylation (f). (g) Dot plot of significantly enriched KEGG pathways identified by GSEA (FDR < 0.05). (h) Dot plot of significantly enriched Gene Ontology (GO) terms based on GSEA (FDR < 0.05).

Fig. S6 Gut microbiome profiles in DSS-induced colitis mice with or without NMN administration. 16S rRNA gene sequencing was performed using four independent biological replicates per group (n = 4). Fecal samples from DSS-induced colitis mice (DSS and DSS + NMN) were analyzed by 16S rRNA gene sequencing. Taxonomic assignment was primarily performed using the GTDB database. Taxa that could not classified by GTDB additionally identified using the SILVA 138 database. (a) Stacked bar plot showing the relative abundance of bacterial phyla (mean across samples). (b) Volcano plot of differential taxonomic abundance based on ANCOM-BC2 analysis. Taxa with significantly increased abundance in NMN-treated mice are shown in red, whereas taxa with decreased abundance are shown in blue (|log2FC| > 0.585, FDR < 0.05). (c) Heatmap of taxa identified as significantly altered by ANCOM-BC2. Relative abundance values are shown, with red and blue indicating relatively higher and lower abundance, respectively. (d, e) Alpha diversity (Chao1, Faith's phylogenetic diversity, observed features, Shannon index, and Simpson index) and principal coordinates analysis (PCoA) based on Bray–Curtis distances in DSS and DSS + NMN mice.

Fig. S7 Quality assessment of RNA-seq data from the long-term NMN administration experiment. RNA sequencing was performed using five independent biological replicates per group (n = 5). (a) Quality assessment of RNA-seq samples by principal component analysis (PCA). PCA was performed using normalized logCPM values from all RNA-seq samples from the two experimental groups (untreated control and NMN-treated mice). (b) Correlation heatmap showing Pearson correlation coefficients among all RNA-seq samples.

Fig. S8 Tumor number in male mice with AOM/DSS-induced colitis-associated cancer (CAC). (a) Representative gross images of colons from AOM/DSS-treated mice (CAC) and AOM/DSS-treated mice receiving long-term NMN administration (CAC + NMN). Arrowheads indicate colonic tumors. (b) Quantification of colonic tumor number (CAC, n = 9; CAC + NMN, n = 10). Data are presented as mean ± SEM. Statistical significance was determined using an unpaired Student's t-test.

Fig. S9 Quality assessment of RNA-seq data from the CAC model. Tumor and adjacent non-tumor tissues were collected from AOM/DSS-treated mice with or without long-term NMN administration. RNA sequencing was performed using five independent biological replicates per group (n = 5). (a) Quality assessment of RNA-seq samples by principal component analysis (PCA). PCA was performed using normalized logCPM values from all RNA-seq samples from the following five groups: untreated control colon tissue, Non-tumor, Non-tumor + NMN, Tumor, and Tumor + NMN. (b) Correlation heatmap showing Pearson correlation coefficients among all RNA-seq samples.

Fig. S10 Immune modulation by NMN in the colitis-associated cancer (CAC) model. RNA-seq analyses were performed using the dataset whose quality assessment is shown in Supplementary Fig. S9. RNA sequencing was performed using five independent biological replicates per group (n = 5). Transcriptomic profiles of colonic tumors from AOM/DSS-treated mice were compared with those from mice receiving AOM/DSS plus long-term NMN administration (100 days). Differentially expressed genes (|log2FC| > 0.585, logCPM > 2, FDR < 0.05) are shown. (a) Volcano plot of differentially expressed genes. Upregulated and downregulated genes are highlighted in light red and sky blue, respectively. (b) Heatmap of the 15 differentially expressed genes between AOM/DSS and AOM/DSS + NMN groups. Gene expression values were scaled by row (z-score). Red and blue indicate relatively higher and lower expression levels, respectively. (c) Relative abundance of multiple cell types estimated by digital deconvolution of bulk RNA-seq data using CIBERSORTx, including epithelial, stromal, and immune cell populations (B cells, colon epithelial cells, endothelial cells, fibroblasts, granulocytes, lymphocytes, macrophages, mast cells, and monocytes). (d) Dot plot of significantly enriched KEGG pathways identified by gene set enrichment analysis (GSEA) (FDR < 0.05). (e) Dot plot of significantly enriched Gene Ontology (GO) terms identified by GSEA (FDR < 0.05). (f, g) Representative GSEA enrichment plots of the KEGG pathways significantly upregulated by NMN administration in the AOM/DSS-induced colitis-associated cancer model, including Antigen processing and presentation (f) and Cellular senescence (g).

Fig. S11 Gene expression changes in colonic tumors from the colitis-associated cancer (CAC) model. RNA-seq analyses were performed using the dataset whose quality assessment is shown in Supplementary Fig. S9. RNA sequencing was performed using five independent biological replicates per group (n = 5). Comparison of gene expression between colonic tumors and adjacent non-tumor tissues from AOM/DSS-treated mice identified 1383 upregulated and 1474 downregulated genes (|log2FC| > 0.585, logCPM > 2, FDR < 0.05). (a) Volcano plot of differential gene expression between tumor and adjacent non-tumor tissues. Genes previously identified as commonly regulated in TGF-β-stimulated MEFs are highlighted, with commonly upregulated genes shown in red and commonly downregulated genes in blue. (b) Heatmap of the top 30 differentially expressed genes. Gene expression values were scaled by row (z-score). Red and blue indicate relatively higher and lower expression levels, respectively. (c) Representative GSEA enrichment plot of the KEGG pathway Basal cell carcinoma, showing positive enrichment in tumor tissues compared with adjacent non-tumor tissues. (d) Dot plot of significantly enriched KEGG pathways identified by GSEA (FDR < 0.05). (e) Dot plot of significantly enriched Gene Ontology (GO) terms identified by GSEA (FDR < 0.05).

Fig. S12 Tumor number in female mice with AOM/DSS-induced colitis-associated cancer (CAC). (a) Representative gross images of colons from AOM/DSS-treated mice (CAC) and AOM/DSS-treated mice receiving long-term NMN administration (CAC + NMN). Arrowheads indicate colonic tumors. (b) Quantification of colonic tumor number (CAC, n = 7; CAC + NMN, n = 10). Data are presented as mean ± SEM. Statistical significance was determined using an unpaired Student's t-test.
