# Supplementary material for: NAD+ metabolism influences TGF-β–associated transcriptional programs and the gut microbiota in murine models of colitis
Source: Biochem Biophys Rep. 2026 Aug 22;47:102752. doi: 10.1016/j.bbrep.2026.102752 (PMC13505500; doi:10.1016/j.bbrep.2026.102752)
Supplement: Supplementary file 1 [file mmc1.pdf]

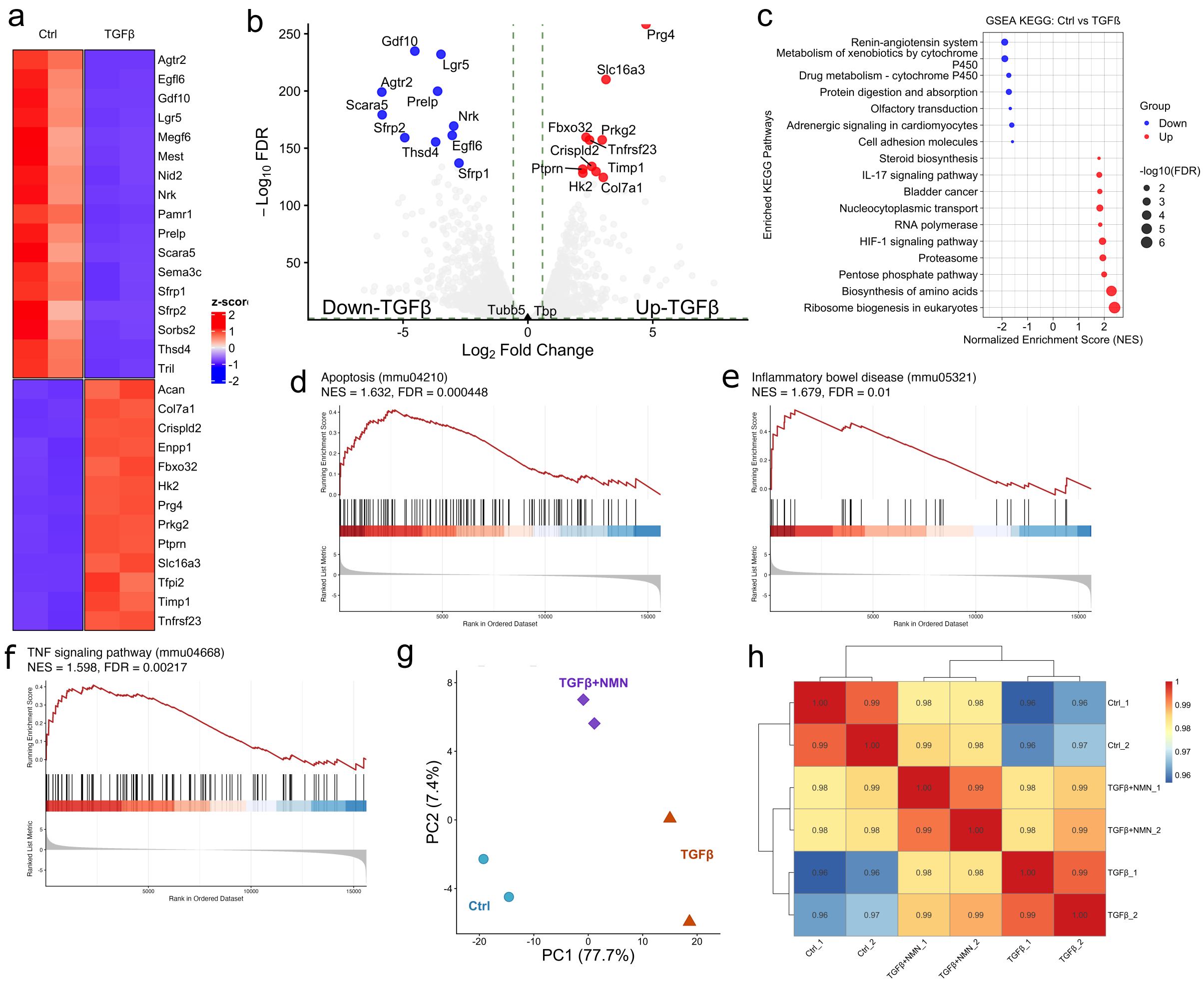

a
Ctrl
TGFβ
Agtr2
Egfl6
Gdf10
Lgr5
Megf6
Mest
Nid2
Nrk
Pamr1
Prelp
Scara5
Sema3c
Sfrp1
Sfrp2
Sorbs2
Thsd4
Tril
Acan
Col7a1
Crispld2
Enpp1
Fbxo32
Hk2
Prg4
Prkg2
Ptprn
Slc16a3
Tfpi2
Timp1
Tnfrsf23
z-score
b
Gdf10
Lgr5
Agtr2
Scara5
Prelp
Nrk
Sfrp2
Egfl6
Thsd4
Sfrp1
Prg4
Slc16a3
Fbxo32
Prkg2
Crispld2
Tnfrsf23
Ptprn
Timp1
Hk2
Col7a1
Down-TGFβ
Up-TGFβ
Tubb5
Tbp
−Log10 FDR
Log2 Fold Change
c
GSEA KEGG: Ctrl vs TGFß
Renin-angiotensin system
Metabolism of xenobiotics by cytochrome P450
Drug metabolism - cytochrome P450
Protein digestion and absorption
Olfactory transduction
Adrenergic signaling in cardiomyocytes
Cell adhesion molecules
Steroid biosynthesis
IL-17 signaling pathway
Bladder cancer
Nucleocytoplasmic transport
RNA polymerase
HIF-1 signaling pathway
Proteasome
Pentose phosphate pathway
Biosynthesis of amino acids
Ribosome biogenesis in eukaryotes
Enriched KEGG Pathways
Normalized Enrichment Score (NES)
Group
Down
Up
-log10(FDR)
d
Apoptosis (mmu04210)
NES = 1.632, FDR = 0.000448
Running Enrichment Score
Ranked List Metric
Rank in Ordered Dataset
e
Inflammatory bowel disease (mmu05321)
NES = 1.679, FDR = 0.01
f
TNF signaling pathway (mmu04668)
NES = 1.598, FDR = 0.00217
g
TGFβ+NMN
TGFβ
Ctrl
PC2 (7.4%)
PC1 (77.7%)
h
Ctrl_1
Ctrl_2
TGFβ+NMN_1
TGFβ+NMN_2
TGFβ_1
TGFβ_2
