# Supplementary material for: NAD+ metabolism influences TGF-β–associated transcriptional programs and the gut microbiota in murine models of colitis
Source: Biochem Biophys Rep. 2026 Aug 22;47:102752. doi: 10.1016/j.bbrep.2026.102752 (PMC13505500; doi:10.1016/j.bbrep.2026.102752)
Supplement: Supplementary file 2 [file mmc10.pdf]

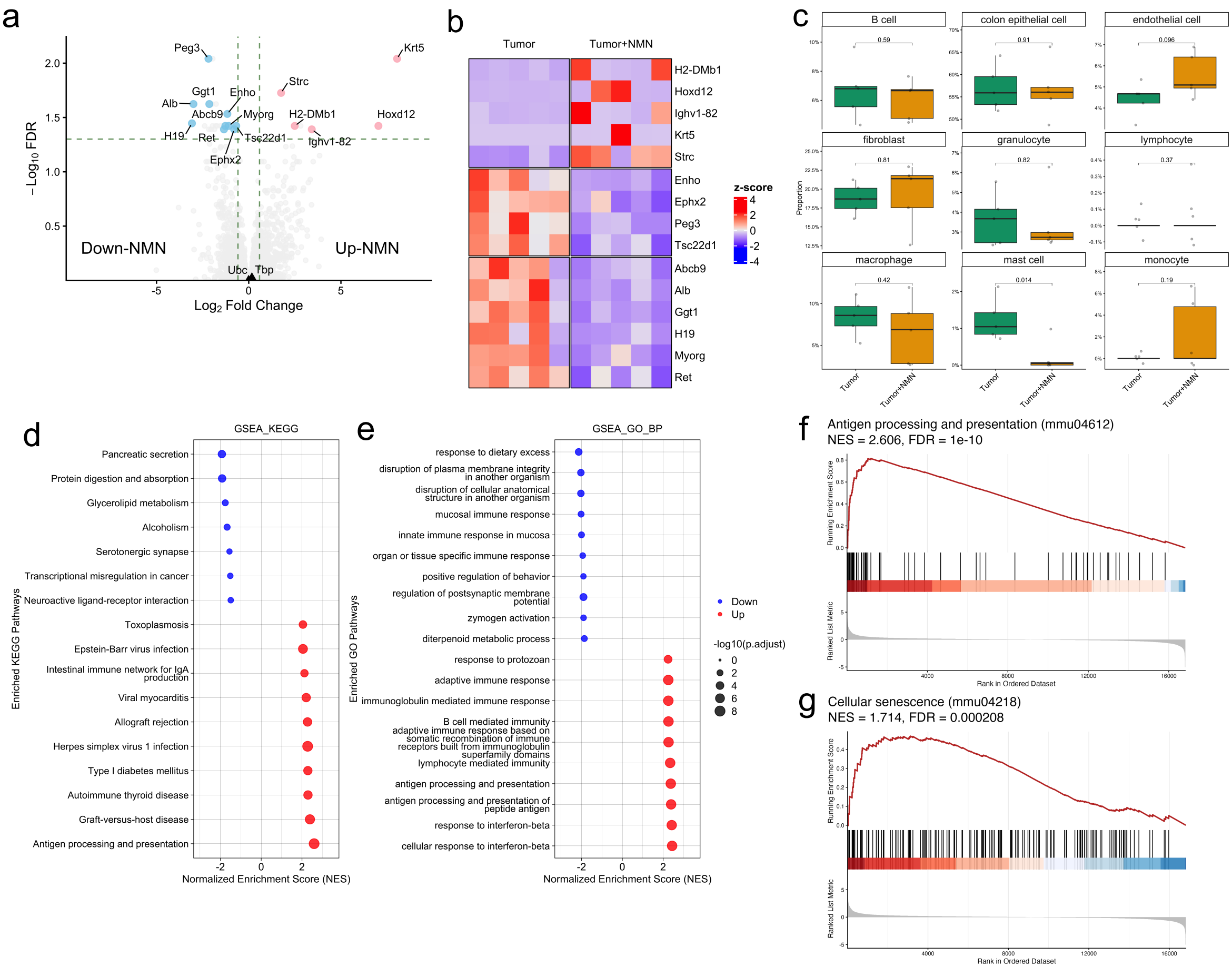

a
Peg3
Krt5
Strc
Ggt1
Enho
Alb
Abcb9
Myorg
H2-DMb1
Hoxd12
H19
Ret
Tsc22d1
Ighv1-82
Ephx2
Down-NMN
Up-NMN
Ubc
Tbp
−Log10 FDR
Log2 Fold Change
b
Tumor
Tumor+NMN
z-score
c
B cell
colon epithelial cell
endothelial cell
fibroblast
granulocyte
lymphocyte
macrophage
mast cell
monocyte
Proportion
Tumor
Tumor+NMN
d
GSEA_KEGG
Pancreatic secretion
Protein digestion and absorption
Glycerolipid metabolism
Alcoholism
Serotonergic synapse
Transcriptional misregulation in cancer
Neuroactive ligand-receptor interaction
Toxoplasmosis
Epstein-Barr virus infection
Intestinal immune network for IgA production
Viral myocarditis
Allograft rejection
Herpes simplex virus 1 infection
Type I diabetes mellitus
Autoimmune thyroid disease
Graft-versus-host disease
Antigen processing and presentation
Enriched KEGG Pathways
Normalized Enrichment Score (NES)
e
GSEA_GO_BP
response to dietary excess
disruption of plasma membrane integrity in another organism
disruption of cellular anatomical structure in another organism
mucosal immune response
innate immune response in mucosa
organ or tissue specific immune response
positive regulation of behavior
regulation of postsynaptic membrane potential
zymogen activation
diterpenoid metabolic process
response to protozoan
adaptive immune response
immunoglobulin mediated immune response
B cell mediated immunity
adaptive immune response based on somatic recombination of immune receptors built from immunoglobulin superfamily domains
lymphocyte mediated immunity
antigen processing and presentation
antigen processing and presentation of peptide antigen
response to interferon-beta
cellular response to interferon-beta
Enriched GO Pathways
Normalized Enrichment Score (NES)
Down
Up
-log10(p.adjust)
f
Antigen processing and presentation (mmu04612)
NES = 2.606, FDR = 1e-10
Running Enrichment Score
Ranked List Metric
Rank in Ordered Dataset
g
Cellular senescence (mmu04218)
NES = 1.714, FDR = 0.000208
Running Enrichment Score
Ranked List Metric
Rank in Ordered Dataset
