# Supplementary material for: NAD+ metabolism influences TGF-β–associated transcriptional programs and the gut microbiota in murine models of colitis
Source: Biochem Biophys Rep. 2026 Aug 22;47:102752. doi: 10.1016/j.bbrep.2026.102752 (PMC13505500; doi:10.1016/j.bbrep.2026.102752)
Supplement: Supplementary file 3 [file mmc11.pdf]

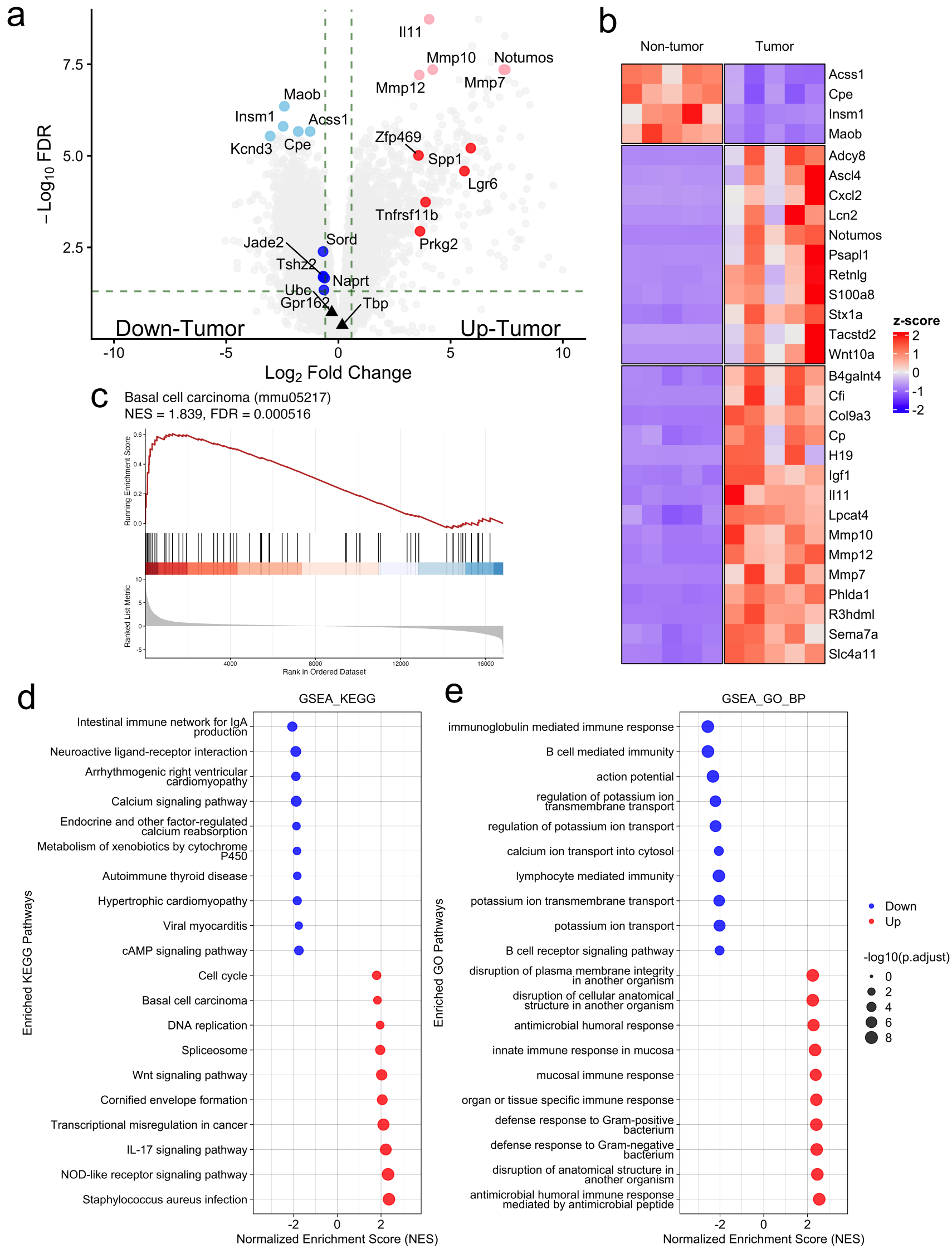
a
Il11
Mmp10
Notumos
Mmp12
Mmp7
Maob
Insm1
Acss1
Kcnd3
Cpe
Zfp469
Spp1
Lgr6
Tnfrsf11b
Prkg2
Jade2
Sord
Tshz2
Naprt
Ubc
Gpr162
Tbp
Down-Tumor
Up-Tumor
−Log10 FDR
Log2 Fold Change
b
Non-tumor
Tumor
Acss1
Cpe
Insm1
Maob
Adcy8
Ascl4
Cxcl2
Lcn2
Notumos
Psapl1
Retnlg
S100a8
Stx1a
Tacstd2
Wnt10a
B4galnt4
Cfi
Col9a3
Cp
H19
Igf1
Il11
Lpcat4
Mmp10
Mmp12
Mmp7
Phlda1
R3hdml
Sema7a
Slc4a11
z-score
c
Basal cell carcinoma (mmu05217)
NES = 1.839, FDR = 0.000516
Running Enrichment Score
Ranked List Metric
Rank in Ordered Dataset
d
GSEA_KEGG
Enriched KEGG Pathways
Intestinal immune network for IgA production
Neuroactive ligand-receptor interaction
Arrhythmogenic right ventricular cardiomyopathy
Calcium signaling pathway
Endocrine and other factor-regulated calcium reabsorption
Metabolism of xenobiotics by cytochrome P450
Autoimmune thyroid disease
Hypertrophic cardiomyopathy
Viral myocarditis
cAMP signaling pathway
Cell cycle
Basal cell carcinoma
DNA replication
Spliceosome
Wnt signaling pathway
Cornified envelope formation
Transcriptional misregulation in cancer
IL-17 signaling pathway
NOD-like receptor signaling pathway
Staphylococcus aureus infection
Normalized Enrichment Score (NES)
e
GSEA_GO_BP
Enriched GO Pathways
immunoglobulin mediated immune response
B cell mediated immunity
action potential
regulation of potassium ion transmembrane transport
regulation of potassium ion transport
calcium ion transport into cytosol
lymphocyte mediated immunity
potassium ion transmembrane transport
potassium ion transport
B cell receptor signaling pathway
disruption of plasma membrane integrity in another organism
disruption of cellular anatomical structure in another organism
antimicrobial humoral response
innate immune response in mucosa
mucosal immune response
organ or tissue specific immune response
defense response to Gram-positive bacterium
defense response to Gram-negative bacterium
disruption of anatomical structure in another organism
antimicrobial humoral immune response mediated by antimicrobial peptide
Normalized Enrichment Score (NES)
Down
Up
-log10(p.adjust)
