# Supplementary material for: NAD+ metabolism influences TGF-β–associated transcriptional programs and the gut microbiota in murine models of colitis
Source: Biochem Biophys Rep. 2026 Aug 22;47:102752. doi: 10.1016/j.bbrep.2026.102752 (PMC13505500; doi:10.1016/j.bbrep.2026.102752)
Supplement: Supplementary file 4 [file mmc12.pdf]

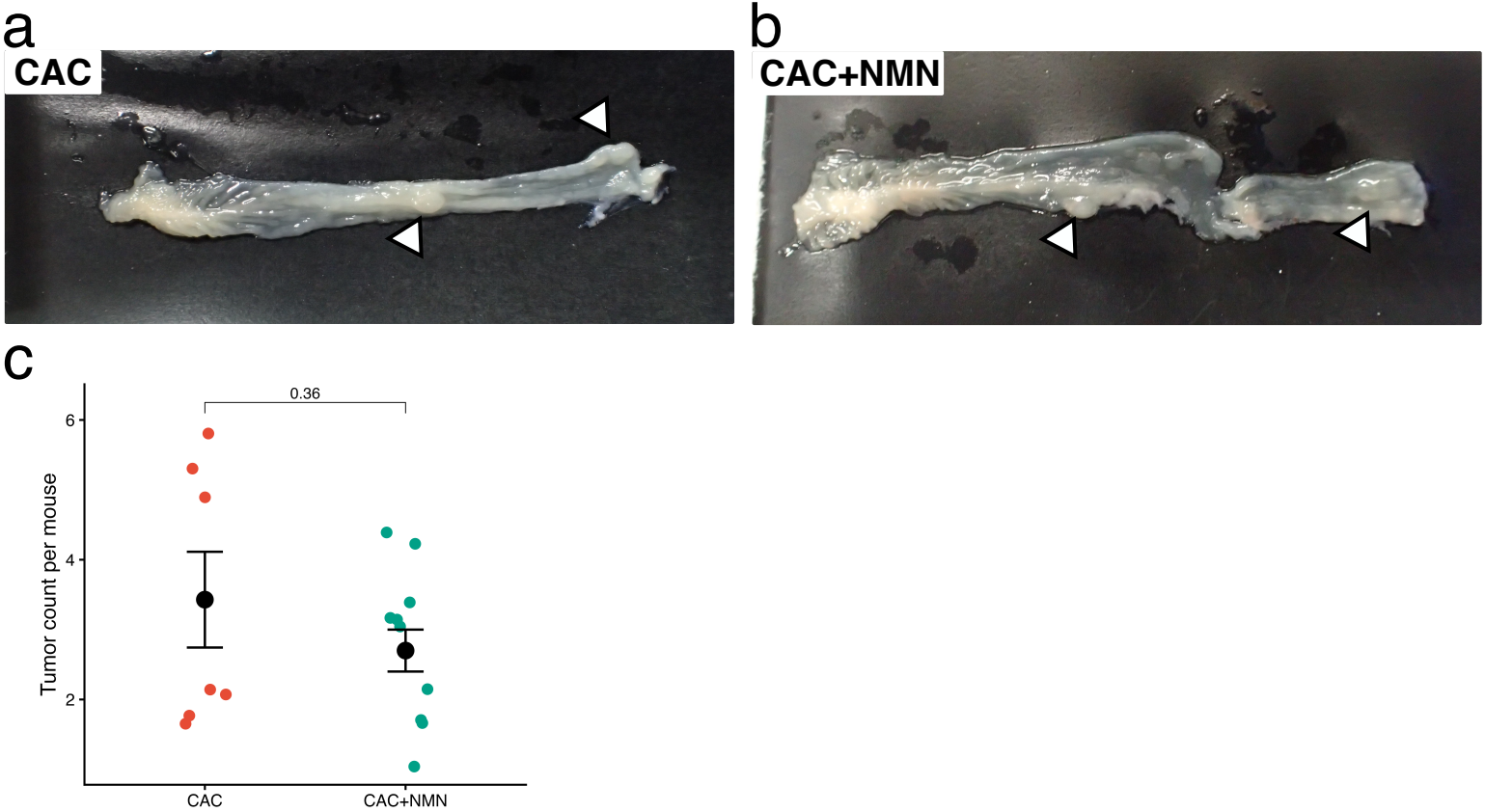

a
CAC
b
CAC+NMN
c
0.36
6
4
2
Tumor count per mouse
CAC
CAC+NMN
