# Supplementary material for: NAD+ metabolism influences TGF-β–associated transcriptional programs and the gut microbiota in murine models of colitis
Source: Biochem Biophys Rep. 2026 Aug 22;47:102752. doi: 10.1016/j.bbrep.2026.102752 (PMC13505500; doi:10.1016/j.bbrep.2026.102752)
Supplement: Supplementary file 5 [file mmc2.pdf]

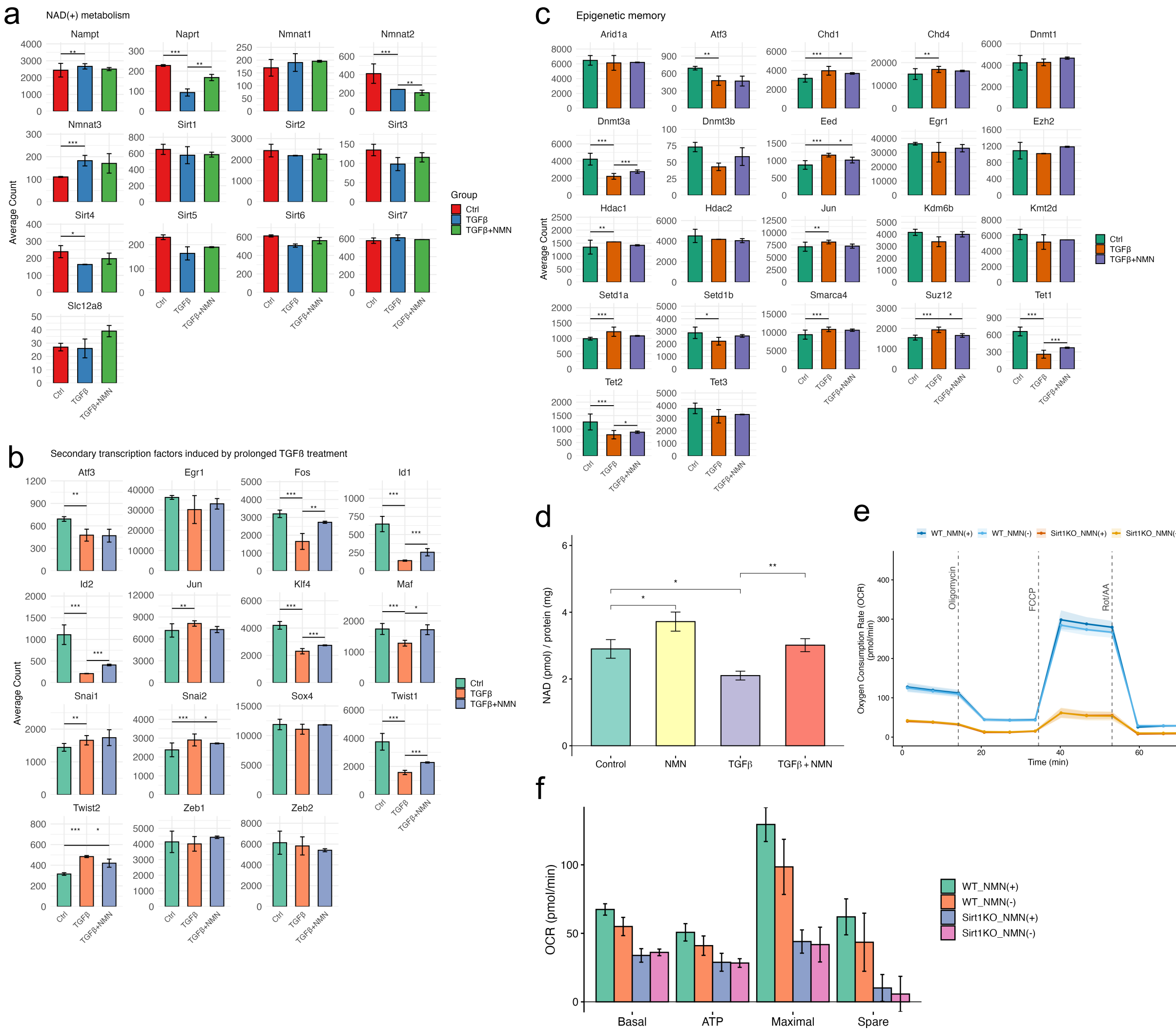
a
NAD(+) metabolism
Nampt
Naprt
Nmnat1
Nmnat2
Nmnat3
Sirt1
Sirt2
Sirt3
Sirt4
Sirt5
Sirt6
Sirt7
Slc12a8
Average Count
Group
Ctrl
TGFβ
TGFβ+NMN
b
Secondary transcription factors induced by prolonged TGFß treatment
Atf3
Egr1
Fos
Id1
Id2
Jun
Klf4
Maf
Snai1
Snai2
Sox4
Twist1
Twist2
Zeb1
Zeb2
Average Count
Ctrl
TGFβ
TGFβ+NMN
c
Epigenetic memory
Arid1a
Atf3
Chd1
Chd4
Dnmt1
Dnmt3a
Dnmt3b
Eed
Egr1
Ezh2
Hdac1
Hdac2
Jun
Kdm6b
Kmt2d
Setd1a
Setd1b
Smarca4
Suz12
Tet1
Tet2
Tet3
Average Count
Ctrl
TGFβ
TGFβ+NMN
d
NAD (pmol) / protein (mg)
Control
NMN
TGFβ
TGFβ + NMN
e
WT_NMN(+)
WT_NMN(-)
Sirt1KO_NMN(+)
Sirt1KO_NMN(-)
Oligomycin
FCCP
Rot/AA
Oxygen Consumption Rate (OCR) (pmol/min)
Time (min)
f
OCR (pmol/min)
Basal
ATP
Maximal
Spare
WT_NMN(+)
WT_NMN(-)
Sirt1KO_NMN(+)
Sirt1KO_NMN(-)
