# Supplementary material for: NAD+ metabolism influences TGF-β–associated transcriptional programs and the gut microbiota in murine models of colitis
Source: Biochem Biophys Rep. 2026 Aug 22;47:102752. doi: 10.1016/j.bbrep.2026.102752 (PMC13505500; doi:10.1016/j.bbrep.2026.102752)
Supplement: Supplementary file 6 [file mmc3.pdf]

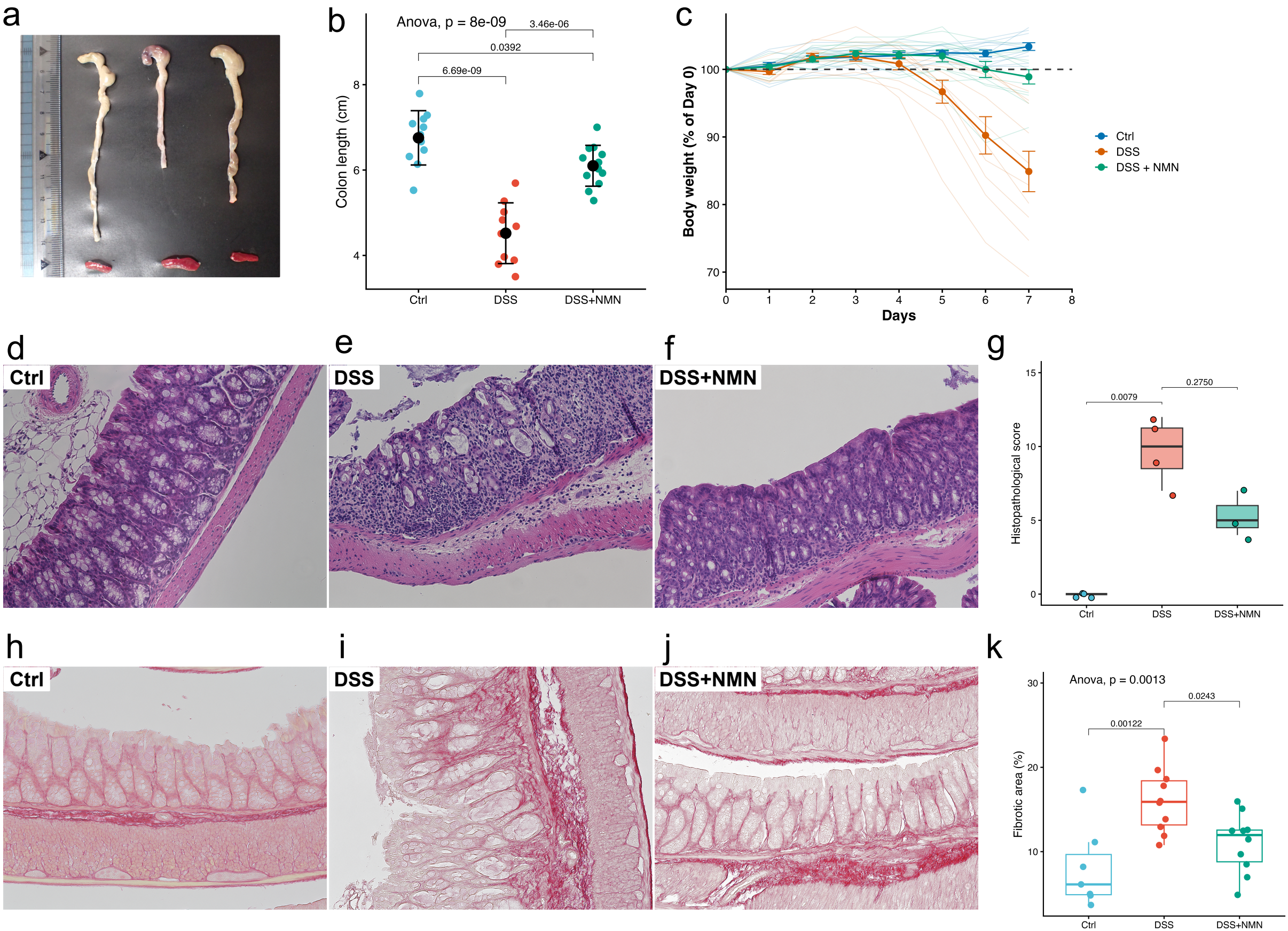
a
b
Anova, p = 8e-09
3.46e-06
0.0392
6.69e-09
Colon length (cm)
Ctrl
DSS
DSS+NMN
c
Body weight (% of Day 0)
Days
Ctrl
DSS
DSS + NMN
d
Ctrl
e
DSS
f
DSS+NMN
g
0.0079
0.2750
Histopathological score
Ctrl
DSS
DSS+NMN
h
Ctrl
i
DSS
j
DSS+NMN
k
Anova, p = 0.0013
0.0243
0.00122
Fibrotic area (%)
Ctrl
DSS
DSS+NMN
