# Supplementary material for: NAD+ metabolism influences TGF-β–associated transcriptional programs and the gut microbiota in murine models of colitis
Source: Biochem Biophys Rep. 2026 Aug 22;47:102752. doi: 10.1016/j.bbrep.2026.102752 (PMC13505500; doi:10.1016/j.bbrep.2026.102752)
Supplement: Supplementary file 7 [file mmc4.pdf]

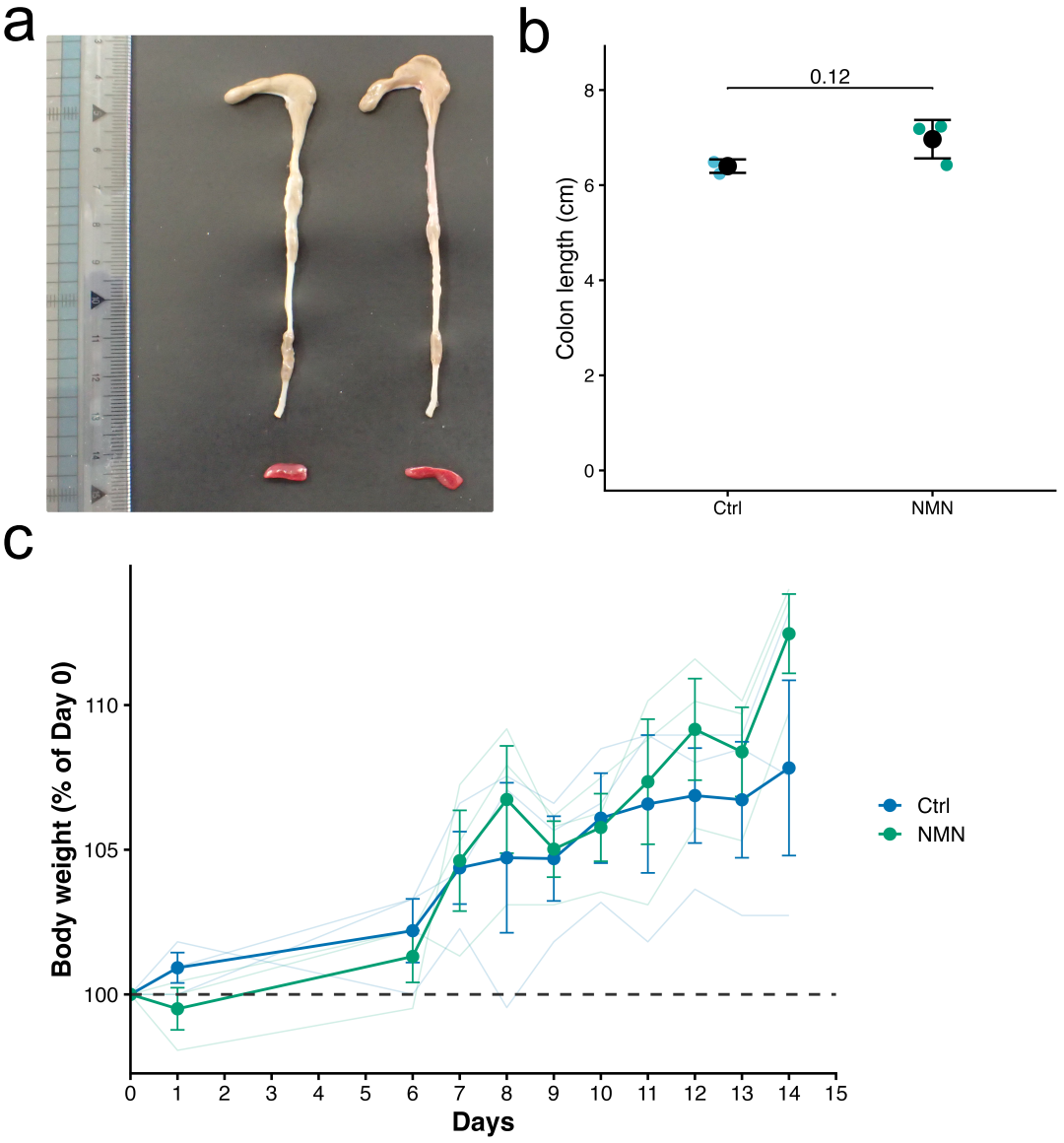

a
b
0.12
Colon length (cm)
0
2
4
6
8
Ctrl
NMN
c
Body weight (% of Day 0)
100
105
110
Days
0
1
2
3
4
5
6
7
8
9
10
11
12
13
14
15
Ctrl
NMN
