# Supplementary material for: NAD+ metabolism influences TGF-β–associated transcriptional programs and the gut microbiota in murine models of colitis
Source: Biochem Biophys Rep. 2026 Aug 22;47:102752. doi: 10.1016/j.bbrep.2026.102752 (PMC13505500; doi:10.1016/j.bbrep.2026.102752)
Supplement: Supplementary file 8 [file mmc5.pdf]

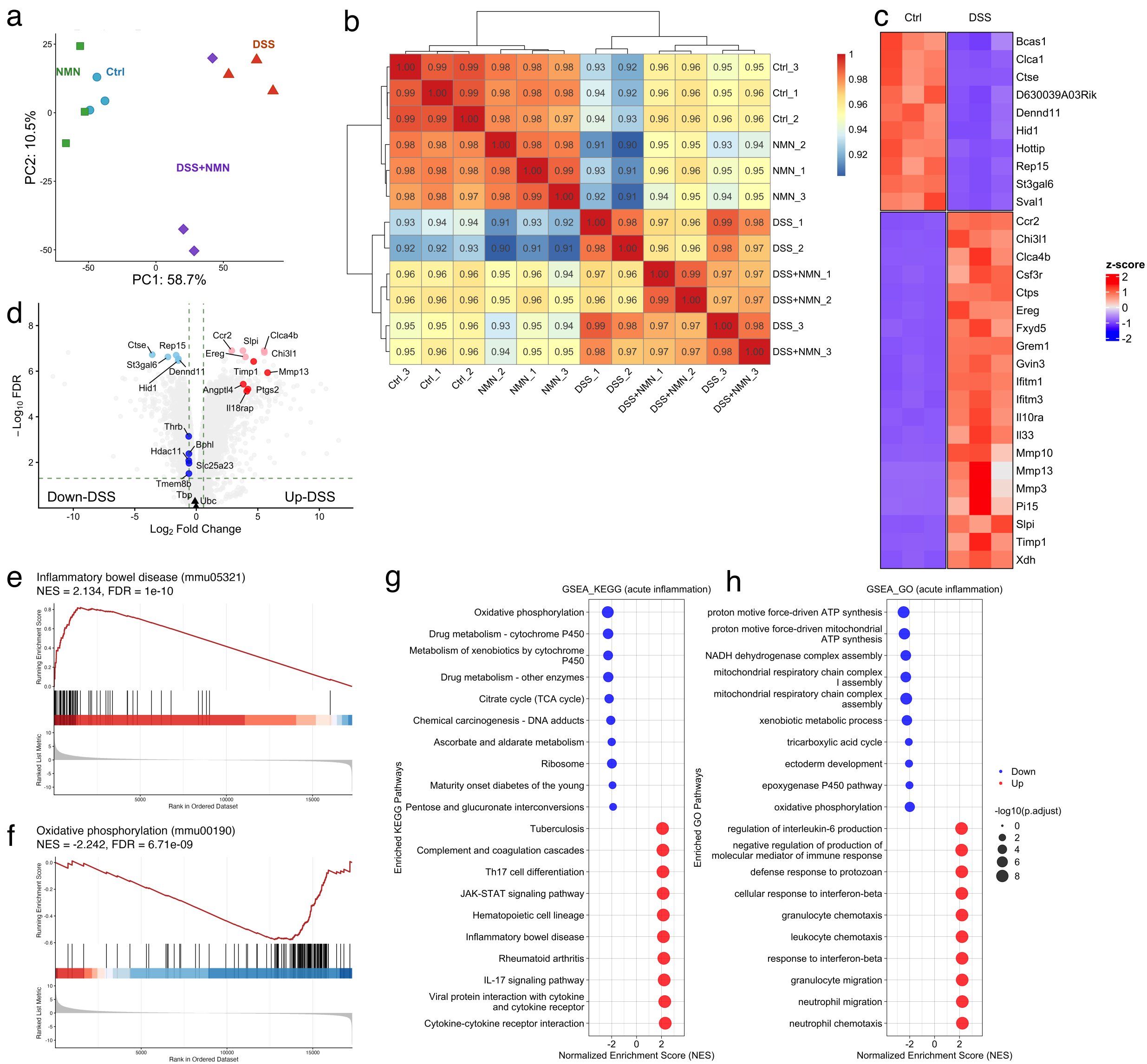

a
NMN
Ctrl
DSS
DSS+NMN
PC2: 10.5%
PC1: 58.7%
b
Ctrl_3 Ctrl_1 Ctrl_2 NMN_2 NMN_1 NMN_3 DSS_1 DSS_2 DSS+NMN_1 DSS+NMN_2 DSS_3 DSS+NMN_3
1.00 0.99 0.99 0.98 0.98 0.98 0.93 0.92 0.96 0.96 0.95 0.95 Ctrl_3
0.99 1.00 0.99 0.98 0.98 0.98 0.94 0.92 0.96 0.96 0.95 0.96 Ctrl_1
0.99 0.99 1.00 0.98 0.98 0.97 0.94 0.93 0.96 0.96 0.96 0.96 Ctrl_2
0.98 0.98 0.98 1.00 0.98 0.98 0.91 0.90 0.95 0.95 0.93 0.94 NMN_2
0.98 0.98 0.98 0.98 1.00 0.99 0.93 0.91 0.96 0.96 0.95 0.95 NMN_1
0.98 0.98 0.97 0.98 0.99 1.00 0.92 0.91 0.94 0.95 0.94 0.95 NMN_3
0.93 0.94 0.94 0.91 0.93 0.92 1.00 0.98 0.97 0.96 0.99 0.98 DSS_1
0.92 0.92 0.93 0.90 0.91 0.91 0.98 1.00 0.96 0.96 0.98 0.97 DSS_2
0.96 0.96 0.96 0.95 0.96 0.94 0.97 0.96 1.00 0.99 0.97 0.97 DSS+NMN_1
0.96 0.96 0.96 0.95 0.96 0.95 0.96 0.96 0.99 1.00 0.97 0.97 DSS+NMN_2
0.95 0.95 0.96 0.93 0.95 0.94 0.99 0.98 0.97 0.97 1.00 0.98 DSS_3
0.95 0.96 0.96 0.94 0.95 0.95 0.98 0.97 0.97 0.97 0.98 1.00 DSS+NMN_3
c
Ctrl
DSS
Bcas1
Clca1
Ctse
D630039A03Rik
Dennd11
Hid1
Hottip
Rep15
St3gal6
Sval1
Ccr2
Chi3l1
Clca4b
Csf3r
Ctps
Ereg
Fxyd5
Grem1
Gvin3
Ifitm1
Ifitm3
Il10ra
Il33
Mmp10
Mmp13
Mmp3
Pi15
Slpi
Timp1
Xdh
z-score
d
Ctse
Rep15
St3gal6
Dennd11
Hid1
Ccr2
Slpi
Clca4b
Ereg
Chi3l1
Timp1
Mmp13
Angptl4
Ptgs2
Il18rap
Thrb
Bphl
Hdac11
Slc25a23
Tmem8b
Tbp
Ubc
Down-DSS
Up-DSS
$-\text{Log}_{10}$ FDR
Log_2 Fold Change
e
Inflammatory bowel disease (mmu05321)
NES = 2.134, FDR = 1e-10
Running Enrichment Score
Ranked List Metric
Rank in Ordered Dataset
f
Oxidative phosphorylation (mmu00190)
NES = -2.242, FDR = 6.71e-09
Running Enrichment Score
Ranked List Metric
Rank in Ordered Dataset
g
GSEA_KEGG (acute inflammation)
Oxidative phosphorylation
Drug metabolism - cytochrome P450
Metabolism of xenobiotics by cytochrome P450
Drug metabolism - other enzymes
Citrate cycle (TCA cycle)
Chemical carcinogenesis - DNA adducts
Ascorbate and aldarate metabolism
Ribosome
Maturity onset diabetes of the young
Pentose and glucuronate interconversions
Tuberculosis
Complement and coagulation cascades
Th17 cell differentiation
JAK-STAT signaling pathway
Hematopoietic cell lineage
Inflammatory bowel disease
Rheumatoid arthritis
IL-17 signaling pathway
Viral protein interaction with cytokine and cytokine receptor
Cytokine-cytokine receptor interaction
Enriched KEGG Pathways
Normalized Enrichment Score (NES)
h
GSEA_GO (acute inflammation)
proton motive force-driven ATP synthesis
proton motive force-driven mitochondrial ATP synthesis
NADH dehydrogenase complex assembly
mitochondrial respiratory chain complex I assembly
mitochondrial respiratory chain complex assembly
xenobiotic metabolic process
tricarboxylic acid cycle
ectoderm development
epoxygenase P450 pathway
oxidative phosphorylation
regulation of interleukin-6 production
negative regulation of production of molecular mediator of immune response
defense response to protozoan
cellular response to interferon-beta
granulocyte chemotaxis
leukocyte chemotaxis
response to interferon-beta
granulocyte migration
neutrophil migration
neutrophil chemotaxis
Enriched GO Pathways
Normalized Enrichment Score (NES)
Down
Up
-log10(p.adjust)
