# Supplementary material for: NAD+ metabolism influences TGF-β–associated transcriptional programs and the gut microbiota in murine models of colitis
Source: Biochem Biophys Rep. 2026 Aug 22;47:102752. doi: 10.1016/j.bbrep.2026.102752 (PMC13505500; doi:10.1016/j.bbrep.2026.102752)
Supplement: Supplementary file 9 [file mmc6.pdf]

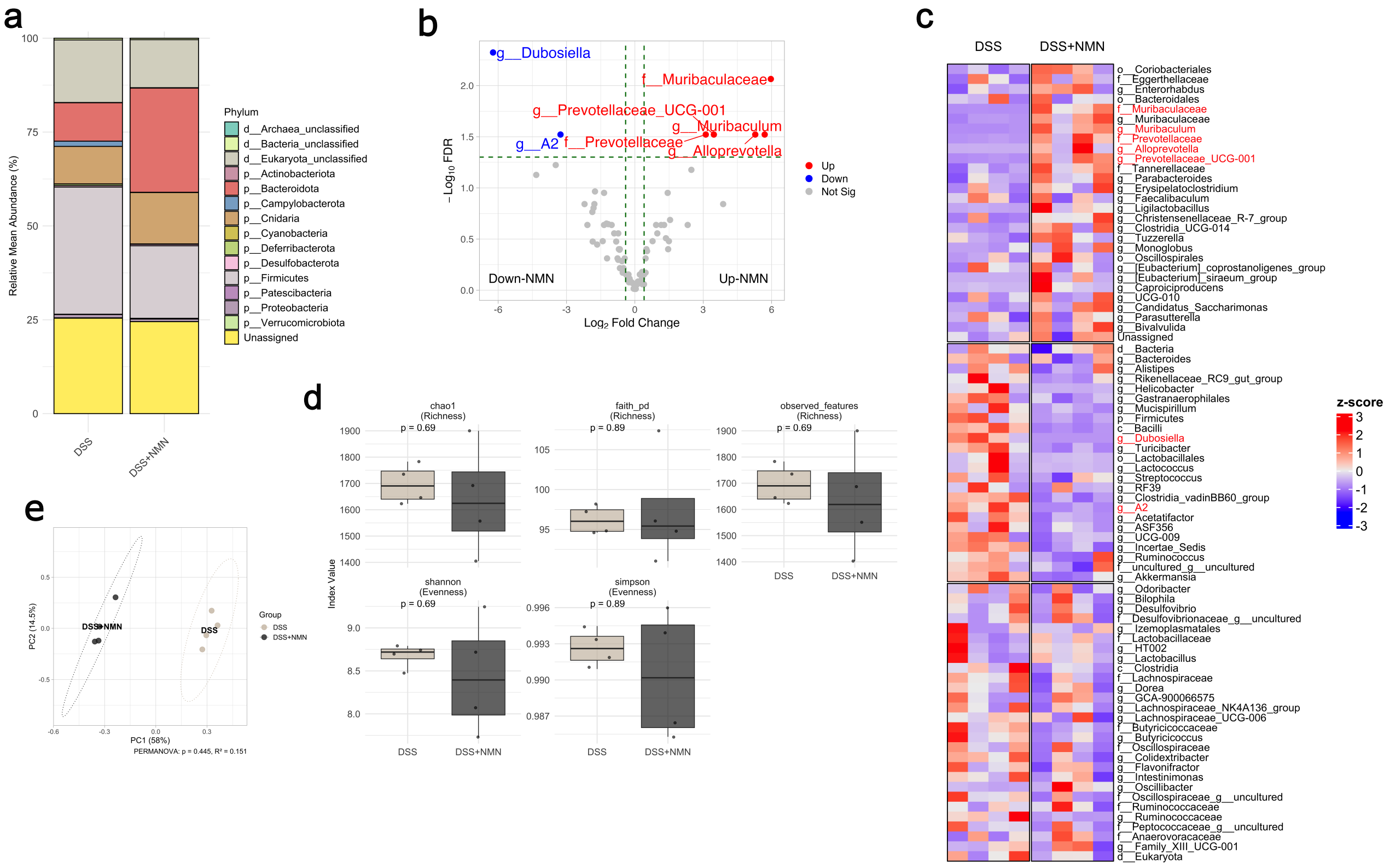
a
Relative Mean Abundance (%)
100
75
50
25
0
DSS
DSS+NMN
Phylum
d__Archaea_unclassified
d__Bacteria_unclassified
d__Eukaryota_unclassified
p__Actinobacteriota
p__Bacteroidota
p__Campylobacterota
p__Cnidaria
p__Cyanobacteria
p__Deferribacterota
p__Desulfobacterota
p__Firmicutes
p__Patescibacteria
p__Proteobacteria
p__Verrucomicrobiota
Unassigned
b
g__Dubosiella
f__Muribaculaceae
g__Prevotellaceae_UCG-001
g__Muribaculum
g__A2
f__Prevotellaceae
g__Alloprevotella
−Log10 FDR
Log2 Fold Change
Down-NMN
Up-NMN
Up
Down
Not Sig
c
DSS
DSS+NMN
o__Coriobacteriales
f__Eggerthellaceae
g__Enterorhabdus
o__Bacteroidales
f__Muribaculaceae
g__Muribaculaceae
g__Muribaculum
f__Prevotellaceae
g__Alloprevotella
g__Prevotellaceae_UCG-001
f__Tannerellaceae
g__Parabacteroides
g__Erysipelatoclostridium
g__Faecalibaculum
g__Ligilactobacillus
g__Christensenellaceae_R-7_group
g__Clostridia_UCG-014
g__Tuzzerella
g__Monoglobus
o__Oscillospirales
g__[Eubacterium]_coprostanoligenes_group
g__[Eubacterium]_siraeum_group
g__Caproiciproducens
g__UCG-010
g__Candidatus_Saccharimonas
g__Parasutterella
g__Bivalvulida
Unassigned
d__Bacteria
g__Bacteroides
g__Alistipes
g__Rikenellaceae_RC9_gut_group
g__Helicobacter
g__Gastranaerophilales
g__Mucispirillum
p__Firmicutes
c__Bacilli
g__Dubosiella
g__Turicibacter
o__Lactobacillales
g__Lactococcus
g__Streptococcus
g__RF39
g__Clostridia_vadinBB60_group
g__A2
g__Acetatifactor
g__ASF356
g__UCG-009
g__Incertae_Sedis
g__Ruminococcus
f__uncultured_g__uncultured
g__Akkermansia
g__Odoribacter
g__Bilophila
g__Desulfovibrio
f__Desulfovibrionaceae_g__uncultured
g__Izemoplasmatales
f__Lactobacillaceae
g__HT002
g__Lactobacillus
c__Clostridia
f__Lachnospiraceae
g__Dorea
g__GCA-900066575
g__Lachnospiraceae_NK4A136_group
g__Lachnospiraceae_UCG-006
f__Butyricicoccaceae
g__Butyricicoccus
f__Oscillospiraceae
g__Colidextribacter
g__Flavonifractor
g__Intestinimonas
g__Oscillibacter
f__Oscillospiraceae_g__uncultured
f__Ruminococcaceae
g__Ruminococcaceae
f__Peptococcaceae_g__uncultured
f__Anaerovoracaceae
g__Family_XIII_UCG-001
d__Eukaryota
z-score
d
chao1 (Richness)
p = 0.69
faith_pd (Richness)
p = 0.89
observed_features (Richness)
p = 0.69
shannon (Evenness)
p = 0.69
simpson (Evenness)
p = 0.89
Index Value
DSS
DSS+NMN
e
PC1 (58%)
PC2 (14.5%)
DSS+NMN
DSS
Group
PERMANOVA: p = 0.445, R² = 0.151
