# Supplementary material for: NAD+ metabolism influences TGF-β–associated transcriptional programs and the gut microbiota in murine models of colitis
Source: Biochem Biophys Rep. 2026 Aug 22;47:102752. doi: 10.1016/j.bbrep.2026.102752 (PMC13505500; doi:10.1016/j.bbrep.2026.102752)
Supplement: Supplementary file 10 [file mmc7.pdf]

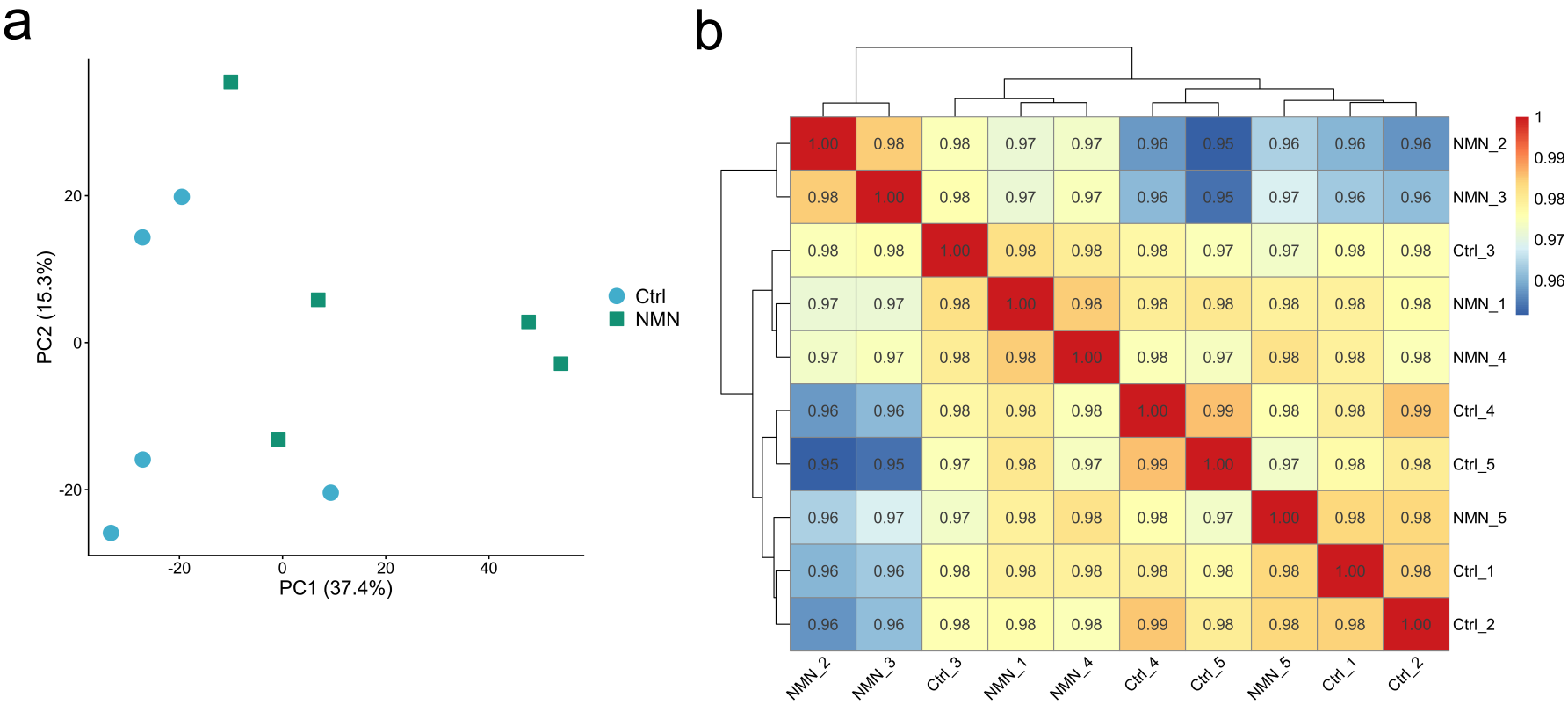
a
PC2 (15.3%)
PC1 (37.4%)
20
0
-20
-20
0
20
40
Ctrl
NMN
b
1
0.99
0.98
0.97
0.96
NMN_2 1.00 0.98 0.98 0.97 0.97 0.96 0.95 0.96 0.96 0.96
NMN_3 0.98 1.00 0.98 0.97 0.97 0.96 0.95 0.97 0.96 0.96
Ctrl_3 0.98 0.98 1.00 0.98 0.98 0.98 0.97 0.97 0.98 0.98
NMN_1 0.97 0.97 0.98 1.00 0.98 0.98 0.98 0.98 0.98 0.98
NMN_4 0.97 0.97 0.98 0.98 1.00 0.98 0.97 0.98 0.98 0.98
Ctrl_4 0.96 0.96 0.98 0.98 0.98 1.00 0.99 0.98 0.98 0.99
Ctrl_5 0.95 0.95 0.97 0.98 0.97 0.99 1.00 0.97 0.98 0.98
NMN_5 0.96 0.97 0.97 0.98 0.98 0.98 0.97 1.00 0.98 0.98
Ctrl_1 0.96 0.96 0.98 0.98 0.98 0.98 0.98 0.98 1.00 0.98
Ctrl_2 0.96 0.96 0.98 0.98 0.98 0.99 0.98 0.98 0.98 1.00
NMN_2
NMN_3
Ctrl_3
NMN_1
NMN_4
Ctrl_4
Ctrl_5
NMN_5
Ctrl_1
Ctrl_2
