# Supplementary material for: NAD+ metabolism influences TGF-β–associated transcriptional programs and the gut microbiota in murine models of colitis
Source: Biochem Biophys Rep. 2026 Aug 22;47:102752. doi: 10.1016/j.bbrep.2026.102752 (PMC13505500; doi:10.1016/j.bbrep.2026.102752)
Supplement: Supplementary file 12 [file mmc9.pdf]

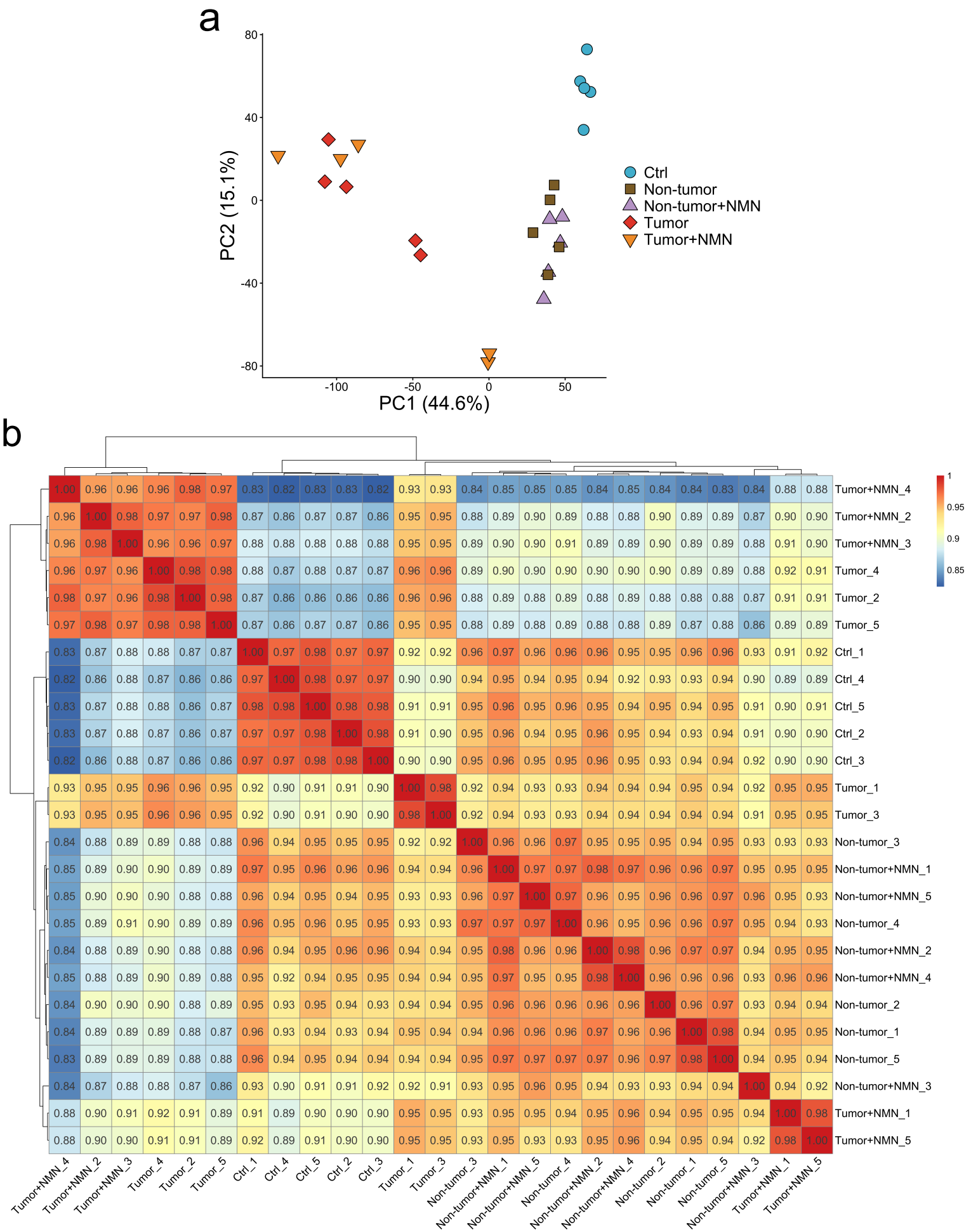
a
80
40
0
-40
-80
-100
-50
0
50
PC2 (15.1%)
PC1 (44.6%)
Ctrl
Non-tumor
Non-tumor+NMN
Tumor
Tumor+NMN
b
1
0.95
0.9
0.85
1.00 0.96 0.96 0.96 0.98 0.97 0.83 0.82 0.83 0.83 0.82 0.93 0.93 0.84 0.85 0.85 0.85 0.84 0.85 0.84 0.84 0.83 0.84 0.88 0.88 Tumor+NMN_4
0.96 1.00 0.98 0.97 0.97 0.98 0.87 0.86 0.87 0.87 0.86 0.95 0.95 0.88 0.89 0.90 0.89 0.88 0.88 0.90 0.89 0.89 0.87 0.90 0.90 Tumor+NMN_2
0.96 0.98 1.00 0.96 0.96 0.97 0.88 0.88 0.88 0.88 0.88 0.95 0.95 0.89 0.90 0.90 0.91 0.89 0.89 0.90 0.89 0.89 0.88 0.91 0.90 Tumor+NMN_3
0.96 0.97 0.96 1.00 0.98 0.98 0.88 0.87 0.88 0.87 0.87 0.96 0.96 0.89 0.90 0.90 0.90 0.90 0.90 0.90 0.89 0.89 0.88 0.92 0.91 Tumor_4
0.98 0.97 0.96 0.98 1.00 0.98 0.87 0.86 0.86 0.86 0.86 0.96 0.96 0.88 0.89 0.88 0.89 0.88 0.89 0.88 0.88 0.88 0.87 0.91 0.91 Tumor_2
0.97 0.98 0.97 0.98 0.98 1.00 0.87 0.86 0.87 0.87 0.86 0.95 0.95 0.88 0.89 0.88 0.89 0.88 0.88 0.89 0.87 0.88 0.86 0.89 0.89 Tumor_5
0.83 0.87 0.88 0.88 0.87 0.87 1.00 0.97 0.98 0.97 0.97 0.92 0.92 0.96 0.97 0.96 0.96 0.96 0.95 0.95 0.96 0.96 0.93 0.91 0.92 Ctrl_1
0.82 0.86 0.88 0.87 0.86 0.86 0.97 1.00 0.98 0.97 0.97 0.90 0.90 0.94 0.95 0.94 0.95 0.94 0.92 0.93 0.93 0.94 0.90 0.89 0.89 Ctrl_4
0.83 0.87 0.88 0.88 0.86 0.87 0.98 0.98 1.00 0.98 0.98 0.91 0.91 0.95 0.96 0.95 0.96 0.95 0.94 0.95 0.94 0.95 0.91 0.90 0.91 Ctrl_5
0.83 0.87 0.88 0.87 0.86 0.87 0.97 0.97 0.98 1.00 0.98 0.91 0.90 0.95 0.96 0.94 0.95 0.96 0.95 0.94 0.93 0.94 0.91 0.90 0.90 Ctrl_2
0.82 0.86 0.88 0.87 0.86 0.86 0.97 0.97 0.98 0.98 1.00 0.90 0.90 0.95 0.96 0.95 0.95 0.96 0.95 0.93 0.94 0.94 0.92 0.90 0.90 Ctrl_3
0.93 0.95 0.95 0.96 0.96 0.95 0.92 0.90 0.91 0.91 0.90 1.00 0.98 0.92 0.94 0.93 0.93 0.94 0.94 0.94 0.95 0.94 0.92 0.95 0.95 Tumor_1
0.93 0.95 0.95 0.96 0.96 0.95 0.92 0.90 0.91 0.90 0.90 0.98 1.00 0.92 0.94 0.93 0.93 0.94 0.94 0.94 0.94 0.94 0.91 0.95 0.95 Tumor_3
0.84 0.88 0.89 0.89 0.88 0.88 0.96 0.94 0.95 0.95 0.95 0.92 0.92 1.00 0.96 0.96 0.97 0.95 0.95 0.95 0.94 0.95 0.93 0.93 0.93 Non-tumor_3
0.85 0.89 0.90 0.90 0.89 0.89 0.97 0.95 0.96 0.96 0.96 0.94 0.94 0.96 1.00 0.97 0.97 0.98 0.97 0.96 0.96 0.97 0.95 0.95 0.95 Non-tumor+NMN_1
0.85 0.90 0.90 0.90 0.88 0.88 0.96 0.94 0.95 0.94 0.95 0.93 0.93 0.96 0.97 1.00 0.97 0.96 0.95 0.96 0.96 0.97 0.96 0.95 0.93 Non-tumor+NMN_5
0.85 0.89 0.91 0.90 0.89 0.89 0.96 0.95 0.96 0.95 0.95 0.93 0.93 0.97 0.97 0.97 1.00 0.96 0.95 0.96 0.96 0.97 0.95 0.94 0.93 Non-tumor_4
0.84 0.88 0.89 0.90 0.88 0.88 0.96 0.94 0.95 0.96 0.96 0.94 0.94 0.95 0.98 0.96 0.96 1.00 0.98 0.96 0.97 0.97 0.94 0.95 0.95 Non-tumor+NMN_2
0.85 0.88 0.89 0.90 0.89 0.88 0.95 0.92 0.94 0.95 0.95 0.94 0.94 0.95 0.97 0.95 0.95 0.98 1.00 0.96 0.96 0.96 0.93 0.96 0.96 Non-tumor+NMN_4
0.84 0.90 0.90 0.90 0.88 0.89 0.95 0.93 0.95 0.94 0.93 0.94 0.94 0.95 0.96 0.96 0.96 0.96 0.96 1.00 0.96 0.97 0.93 0.94 0.94 Non-tumor_2
0.84 0.89 0.89 0.89 0.88 0.87 0.96 0.93 0.94 0.93 0.94 0.95 0.94 0.94 0.96 0.96 0.96 0.97 0.96 0.96 1.00 0.98 0.94 0.95 0.95 Non-tumor_1
0.83 0.89 0.89 0.89 0.88 0.88 0.96 0.94 0.95 0.94 0.94 0.94 0.94 0.95 0.97 0.97 0.97 0.97 0.96 0.97 0.98 1.00 0.94 0.95 0.94 Non-tumor_5
0.84 0.87 0.88 0.88 0.87 0.86 0.93 0.90 0.91 0.91 0.92 0.92 0.91 0.93 0.95 0.96 0.95 0.94 0.93 0.93 0.94 0.94 1.00 0.94 0.92 Non-tumor+NMN_3
0.88 0.90 0.91 0.92 0.91 0.89 0.91 0.89 0.90 0.90 0.90 0.95 0.95 0.93 0.95 0.95 0.94 0.95 0.96 0.94 0.95 0.95 0.94 1.00 0.98 Tumor+NMN_1
0.88 0.90 0.90 0.91 0.91 0.89 0.92 0.89 0.91 0.90 0.90 0.95 0.95 0.93 0.95 0.93 0.93 0.95 0.96 0.94 0.95 0.94 0.92 0.98 1.00 Tumor+NMN_5
Tumor+NMN_4
Tumor+NMN_2
Tumor+NMN_3
Tumor_4
Tumor_2
Tumor_5
Ctrl_1
Ctrl_4
Ctrl_5
Ctrl_2
Ctrl_3
Tumor_1
Tumor_3
Non-tumor_3
Non-tumor+NMN_1
Non-tumor+NMN_5
Non-tumor_4
Non-tumor+NMN_2
Non-tumor+NMN_4
Non-tumor_2
Non-tumor_1
Non-tumor_5
Non-tumor+NMN_3
Tumor+NMN_1
Tumor+NMN_5
