# Supplementary material for: NAD+ metabolism influences TGF-β–associated transcriptional programs and the gut microbiota in murine models of colitis
Source: Biochem Biophys Rep. 2026 Aug 22;47:102752. doi: 10.1016/j.bbrep.2026.102752 (PMC13505500; doi:10.1016/j.bbrep.2026.102752)
Supplement: Supplementary file 13 [file mmc13.docx]

**Supplementary Table S1. Mapping of sequencing and processing pipelines to each experiment**

| **Experiment** | **Sample type** | **Data type** | **Library preparation** | **Sequencing platform** | **Primary processing** | **Differential expression** | **Functional enrichment** | **Cell-type analysis** | **Reference database** |
| --- | --- | --- | --- | --- | --- | --- | --- | --- | --- |
| MEFs | Mouse embryonic fibroblasts | RNA-seq | TruSeq | HiSeq 2500 | Trim Galore! → HISAT2 → featureCounts | edgeR | clusterProfiler | — | GRCm39, GENCODE vM34 |
| DSS colitis model | Mouse colon tissue | RNA-seq | TruSeq | HiSeq 2500 | Trim Galore! → HISAT2 → featureCounts | edgeR | clusterProfiler | — | GRCm39, GENCODE vM34 |
| NMN long-term administration | Mouse colon tissue | RNA-seq | NEBNext | NovaSeq 6000 | Trim Galore! → HISAT2 → featureCounts | edgeR | clusterProfiler | — | GRCm39, GENCODE vM34 |
| AOM+DSS CAC model | Mouse colon tissue | RNA-seq | NEBNext | NovaSeq 6000 | Trim Galore! → HISAT2 → featureCounts | edgeR | clusterProfiler | CIBERSORTx, Seurat | GRCm39, GENCODE vM34 |
|  |  |  |  |  |  |  |  |  |  |
| **Experiment** | **Sample type** | **Data type** | **Library preparation** | **Sequencing platform** | **Primary processing** | **Statistical analysis** | **Functional profiling** | **Reference database** |  |
| Microbiome | Mouse fecal samples | 16S rRNA sequencing | Ion 16S kit | Ion S5 | QIIME2 | ANCOMBC, ALDEx2, MaAsLin3 | PICRUSt2, DESeq2 | GTDB (primary taxonomy), SILVA v138 (supporting reference) |  |
