# Supplementary material for: NAD+ metabolism influences TGF-β–associated transcriptional programs and the gut microbiota in murine models of colitis
Source: Biochem Biophys Rep. 2026 Aug 22;47:102752. doi: 10.1016/j.bbrep.2026.102752 (PMC13505500; doi:10.1016/j.bbrep.2026.102752)
Supplement: Supplementary file 14 [file mmc14.docx]

| **Supplementary Table S2. Concordance of differential bacterial taxa identified by ANCOM-BC2, ALDEx2, and MaAsLin3 analyses** | | | | |
| --- | --- | --- | --- | --- |
|  |  |  |  |  |
| Taxon | ANCOM_BC2 | ALDEx2 | MaAsLin3 | Direction |
| g__Anaerocolumna | Yes | Yes | Yes | Decreased |
| g__Dubosiella | Yes | Yes | Yes | Decreased |
| g__Alloprevotella | Yes | No | Yes | Increased |
| g__UBA3855 | Yes | No | Yes | Increased |
| c__Bacilli | Yes | Yes | Yes | Decreased |
| g__CAG-465 | Yes | No | Yes | Increased |
| g__Prevotella | Yes | No | Yes | Increased |
| g__Muribaculum | No | No | Yes | Increased |
| f__Muribaculaceae | No | No | Yes | Increased |
| g__RACS-045 | No | Yes | No | Decreased |
| g__Fimimonas | No | Yes | No | Decreased |
| f__Anaerotignaceae | No | Yes | No | Decreased |
| g__Turicibacter | No | Yes | No | Decreased |
| g__Sporofaciens | No | Yes | No | Decreased |
| g__Coproplasma | No | Yes | No | Decreased |
| g__CAG-475 | No | Yes | No | Decreased |
| g__Porcincola | No | Yes | No | Decreased |
| g__Heteroclostridium | No | Yes | No | Decreased |
| g__RGIG8607 | No | No | Yes | Increased |
|  |  |  |  |  |
| **Notes:** Taxonomic levels are shown according to the highest resolved classification available for each feature. Yes indicates significant differential abundance identified by the corresponding method (FDR < 0.05). ALDEx2 significance was determined using the Benjamini–Hochberg adjusted within-group expected p-value (wi.eBH). Direction indicates the change in abundance in the DSS+NMN group compared with the DSS group. | | | | |
